# Cell cycle genes and ovarian cancer susceptibility: a tagSNP analysis

**DOI:** 10.1038/sj.bjc.6605284

**Published:** 2009-09-08

**Authors:** J M Cunningham, R A Vierkant, T A Sellers, C Phelan, D N Rider, M Liebow, J Schildkraut, A Berchuck, F J Couch, X Wang, B L Fridley, Ovarian Cancer Association Consortium, A Gentry-Maharaj, U Menon, E Hogdall, S Kjaer, A Whittemore, R DiCioccio, H Song, S A Gayther, S J Ramus, P D P Pharaoh, E L Goode

**Affiliations:** 1 Department of Laboratory Medicine and Pathology, Mayo Clinic College of Medicine, 200 First Street SW, Rochester, MN 55905, USA; 2 Department of Health Sciences Research, Mayo Clinic College of Medicine, 200 First Street SW, Rochester, MN 55905, USA; 3 Department of Epidemiology and Genetics, H. Lee Moffitt Cancer Center and Research Institute, 12902 Magnolia Drive, Tampa, FL 33612, USA; 4 Department of Internal Medicine, Mayo Clinic College of Medicine, 200 First Street SW, Rochester, MN 55905, USA; 5 Department of Community and Family Medicine, Duke University Medical Center, Durham, NC 27710, USA; 6 Department of Obstetrics and Gynaecology, Duke University Medical Center, Durham, NC 27710, USA; 7 Department of Gynaecological Oncology, UCL EGA Institute for Women's Health, University College London, 46 Cleveland St., London, WIT 4JF, UK; 8 Department of Viruses, Hormones, and Cancer, Institute of Cancer Epidemiology, Danish Cancer Society, Righospitalet Strandboulevarden 49, Copenhagen, DK-2100, Denmark; 9 Department of Gynaecology and Obstetrics, Gynaecologic Clinic, Juliane Marie Center, Rigshospitalet, University of Copenhagen, Copenhagen, CR-UK, Denmark; 10 Department of Health Research and Policy, Stanford University School of Medicine, Palo Alto, CA 94305, USA; 11 Department of Cancer Genetics, Roswell Park Cancer Institute, Buffalo, New York 14263, USA; 12 Department of Oncology, Cancer Research United Kingdom and Strangeways Research Laboratory, Cambridge University, Worts Causeway, CB18RN, Cambridge, UK

**Keywords:** ovarian cancer, cell cycle, tag SNPs genes

## Abstract

**Background:**

Dysregulation of the cell cycle is a hallmark of many cancers including ovarian cancer, a leading cause of gynaecologic cancer mortality worldwide.

**Methods:**

We examined single nucleotide polymorphisms (SNPs) (*n*=288) from 39 cell cycle regulation genes, including cyclins, cyclin-dependent kinases (CDKs) and CDK inhibitors, in a two-stage study. White, non-Hispanic cases (*n*=829) and ovarian cancer-free controls (*n*=941) were genotyped using an Illumina assay.

**Results:**

Eleven variants in nine genes (*ABL1, CCNB2, CDKN1A, CCND3, E2F2, CDK2, E2F3, CDC2*, and *CDK7*) were associated with risk of ovarian cancer in at least one genetic model. Seven SNPs were then assessed in four additional studies with 1689 cases and 3398 controls. Association between risk of ovarian cancer and *ABL1* rs2855192 found in the original population [odds ratio, OR_BB *vs* AA_ 2.81 (1.29–6.09), *P*=0.01] was also observed in a replication population, and the association remained suggestive in the combined analysis [OR_BB *vs* AA_ 1.59 (1.08–2.34), *P*=0.02]. No other SNP associations remained suggestive in the replication populations.

**Conclusion:**

*ABL1* has been implicated in multiple processes including cell division, cell adhesion and cellular stress response. These results suggest that characterization of the function of genetic variation in this gene in other ovarian cancer populations is warranted.

Ovarian cancer is the seventh most common cancer and the fourth leading cause of cancer death in women worldwide (Parkin *et al*, 2005). With the highest mortality of all gynaecological malignancies, 15 520 deaths were estimated in the US in 2008 (American Cancer Society, 2008). The pathogenesis and progression of ovarian cancer is not well understood, which contributes to its poor survival, along with difficulties in early detection among asymptomatic women. Modifiable risk factors, which are few, include oral contraceptives, family history and age at menarche. Known genetic risk factors are restricted to mutations inherited in the high risk, high penetrant genes (e.g. *BRCA1/2* and DNA mismatch repair genes), which are rare in the general population and estimated to account for no greater than 10–15% of ovarian cancer (Chen *et al*, 2006; Lancaster *et al*, 2007). Owing to a consensus that genetic factors have a function in susceptibility to ovarian cancer, studies targeting specific pathways in ovarian cancer case–control studies have emerged (Dicioccio *et al*, 2004; Auranen *et al*, 2005; Beesley *et al*, 2007; Song *et al*, 2007; Mann *et al*, 2008; Pearce *et al*, 2008; Quaye *et al*, 2008) and some report nominally significant associations with ovarian cancer risk (Buller *et al*, 1997; Berchuck *et al*, 2004; Dicioccio *et al*, 2004; Kelemen *et al*, 2008; Pearce *et al*, 2008; Sellers *et al*, 2008).

Dysregulation of the cell cycle is a hallmark of many cancers (Pharoah *et al*, 2007; Butt *et al*, 2008; Nam and Kim, 2008) and control and timing of the cell cycle involves checkpoints and regulatory pathways that ensure the fidelity of DNA replication and chromosome segregation (Elledge, 1996). These processes involve a large collection of key molecules, which are excellent candidates for ovarian cancer susceptibility variants. These include the cyclins (*CCNA1*, *CCNA2*, *CCNB1*, *CCNB2*, *CCND1*, *CCND2*, *CCND3*, *CCNE1*, *CCNE2*, *CCNG1*, *CCNG2*), cyclin-dependent kinases (CDKS: *CDK2*, *CDK4*, *CDK6*, *CDK7*, *CDC2*), CDK inhibitors (*CDKN1A*, *CDKN1B*, *CDKN2A*, *CDKN2B*, *CDKN2C*, *CDKN2D*) and CDC2 regulators (*CDC25A*, *CDC25B*). The catalytic subunit of CDKs is activated by one of many activating subunits, the cyclins. Cyclin levels oscillate during the cell cycle, and cyclin–CDK complexes finely regulate progression through the cell cycle. Inhibitors of CDK promote cell cycle arrest and may affect response to mitogenic stimuli. In addition to the cyclins, CDKs and CDK inhibitors, the E2 family of transcription factors is a critical element as well as the E2F family's dimerization partners TFDP1, TFDP2, CUL1 and SKP2, which are involved in the SCF ubiquitin ligase complex. In addition, Rb (and two Rb-like genes) regulates progression of cells from G1 to S to G2 phases. CCND, CCNE and E2F are over-expressed in a variety of cancer, including ovarian cancer (D′Andrilli *et al*, 2004), and data emanating from an immunohistochemical study of ovarian cancer (Hashiguchi *et al*, 2004) reveals alteration of G2 in ovarian cancer specimens. The SCF ubiquitin ligases are well-characterized mammalian cullin RING ubiquitin ligases (Frescas and Pagano, 2008), and this complex is an essential element in the CDKNA–CDK2 S phase. SKP2 activates CDK2 and CDK1 by directing the degradation of CDKN1 (p27) and CDKN1B (p21). SKP2 is also known to target tumour suppressor proteins p21 and CDKN1C, resulting in protein degradation (Frescas and Pagano, 2008). Activation and inactivation of CDKs is an additional crucial process, and dysregulation may be involved in cell transformation. Other important kinases include ABL1, a non-tyrosine kinase, that may regulate the CDC2 kinase (Lin *et al*, 2004), and PLK1, a cell cycle regulated kinase (Yuan *et al*, 2002).

As cell cycle abnormalities have been observed in ovarian cancer (Milde-Langosch and Riethdorf, 2003; De Meyer *et al*, 2009), we hypothesized that common genetic variation in genes altering the functionality of the molecules may influence the ovarian carcinogenic process. An earlier study of 13 genes (88 informative single nucleotide polymorphisms, SNPs) involved in regulation of the G1–S phase of the cell cycle (*CCNDA*, *CCND2*, *CCND3*, *CCNE1*, *CDK2*, *CDK4*, *CDK6*, *CDKN1A*, *CDKN1B*, *CDKN2A*, *CDKN2B*, *CDKN2C* and *CDKN2D)* found nominally significant associations between SNPs in *CDKN2A* and *CDKN1B* [rs3731257 homozygous minor *vs* homozygous major odds ratio, OR_BB *vs* AA_, 0.87 (95% confidence interval, 95% CI, 0.73–1.03) *P-*value=0.021; rs2066827 OR_BB *vs* AA_ 0.79 (0.66–0.95) *P-*value=0.04] (Gayther *et al*, 2007). In addition, a combined analyses of 6 studies and 12 genes including imputed genotypes found evidence of association with selected SNPs in *CDKN2A*, *CCND1*, *CDK2* and *CCNE1*, but not in *CDKN2C*, *CDKN1A*, *CCND3*, *CCND2*, *CDKN1B*, *CDK4*, *RB1*, *CDKN2D* or *CDKN2B* (Goode *et al*, 2009) Here, we report on a more comprehensive two-stage analysis of the association of ovarian cancer risk at 39 genes (288 SNPs) involved in G1/S and G2/M phases of the cell cycle and transcription- and ubiquitin-mediated degradation (Table 1).

## Materials and methods

This study used a two-stage approach: a discovery set comprised of two populations and a replication set comprised of four additional populations. SNPs with suggestive statistical significance in the discovery set were carried through to the replication set to validate the results. Details for these sets and SNP selection are provided below.

### Discovery set

The discovery population comprised of 2051 women participating in an ongoing ovarian cancer case–control study at the Mayo Clinic (MAY) and Duke University (NCO) recruited between June 1999 and March 2006, as described earlier (Kelemen *et al*, 2008; Sellers *et al*, 2008). Study protocols were approved by the Institutional Review Boards at both institutions, and study participants provided written informed consent. Cases were women for whom a diagnosis of histologically confirmed primary epithelial ovarian cancer was ascertained within 1 year of consent. Information on known and suspected ovarian cancer risk factors was collected by in-person interviews, including race/ethnicity, menstrual and reproductive history, use of exogenous hormones, medical and surgical history, tobacco use levels, education level, height and weight 1 year before interview and family history of breast and ovarian cancer in first- or second-degree relatives. DNA was extracted from fresh peripheral blood using the Gentra AutoPure LS Puregene salting out methodology (Gentra Inc, Minneapolis, MN, USA). For NCO samples with limited DNA available, WGA was performed using the REPLI-G protocol (Qiagen) with 200 ng genomic DNA as input yielding high molecular weight DNA and reproducible genotype data (Cunningham *et al*, 2008). Of the 2051 eligible participants, 1967 (95.6%) were successfully genotyped, including 1770 white, non-Hispanic participants used in this report (829 cases and 941 controls).

### Replication sets

Four case–control study populations were included in a replication analysis: the SEARCH ovarian cancer study from East Anglia, United Kingdom (SEA), the MALOVA cancer study from Denmark (MAL), the GEOCS study from Stanford University in Palo Alto, CA (STA) and the UK OPS Study from the United Kingdom (UKO). The SEA study (696 cases/1227 controls) included invasive epithelial ovarian cancer cases collected from the East Anglian and West Midlands cancer registries, and controls randomly selected from European Prospective Investigation into Cancer and Nutrition (EPIC) – Norfolk cohort study. The MAL study (439 cases/1215 controls) contained invasive ovarian cancer cases and population controls randomly drawn from a defined study area in Denmark. The STA study (285 cases/364 controls) ascertained participants from six counties in northern California including invasive ovarian cancer cases and age-matched controls obtained using random-digit dialling. The UKO study (269 cases/592 controls) drew cases from 10 gynaecologic oncology National Health Service Centers and apparently healthy controls from the UK Collaborating Trial of Ovarian Cancer Screening (UKCTOCS). Additional replication study participant details are provided elsewhere (Gayther *et al*, 2007; Ramus *et al*, 2008). Only white, non-Hispanic participants were included.

### Discovery SNP selection and genotyping

SNP selection for the discovery set involved identifying tagSNPs for the 39 genes (Table 1; Supplemental Table 1). To accomplish this, genotype data from the HapMap consortium http://hapmap.org, Seattle SNPs http://pga.mbt.washington.edu, Perlegen Sciences http://genome.perlegen.com and Panel 2 of the National Institute for Environmental Health Sciences http://egp.gs.washingon.edu were analysed with ldSelect (Carlson *et al*, 2004) to bin SNPs with European American MAF >0.05 at a pairwise linkage disequilibrium (LD) threshold of *r*^2^ ⩾0.8. The region for each gene included 5 kb upstream and downstream. Using these data, 288 tagSNPs and putative functional SNPs (non-synonymous coding SNPs and SNPs altering splicing) for the 39 cell cycle genes were included in one of two genotyping panels consisting of 2688 SNPs as part of a larger genotyping effort (Supplementary Tables S1 and S2). Details about the Illumina GoldenGate genotyping have been reported earlier (Cunningham *et al*, 2008; Kelemen *et al*, 2008; Sellers *et al*, 2008). Illumina design scores were >0.6 for 94.9% of the SNPs. Quality control data for the 288 cell cycle SNPs are provided in Supplementary Table S2. SNP call rates were >0.95 and replicate concordance was >0.99.

### Replication SNP selection and genotyping

SNPs with log-additive *P-*values <0.05 were considered for replication. In addition, for SNPs not selected under the log-additive model, but with a suggestion of association in either dominant or recessive models, a more stringent threshold was applied (*P-*value ⩽0.03) for inclusion in the replication (statistical methods described below). One of these SNPs (*CDK2* rs2069414) could not be genotyped using TaqMan, the replication platform, and one of these SNPs (*CCND3* rs3218086) was replaced by rs3218092, which was in LD (*r*^2^=0.95) with rs3218086 and had earlier been genotyped. Thus, six replication SNPs were genotyped at the Strangeways Research Laboratory using TaqMan designed assays, following the manufacturer's recommended protocols; rs3218092 had been similarly assayed (Gayther *et al*, 2007). Each assay used 10 ng DNA in a 5 *μ*l reaction volume with TaqMan universal PCR Master Mix (Applied Biosystems, Warrington, UK); primer and probe sequences, as well as assay conditions, are available on request. TaqMan Allele Discrimination Sequence Detection software (Applied Biosystems) was used to determine genotype calls. SNP call rates were >0.95 and replicate concordance was >0.99.

### Statistical analyses

Discovery set participants were examined initially and restricted to white, non-Hispanic participants. Departures from Hardy–Weinberg equilibrium (HWE) for each SNP were examined using Pearson goodness-of-fit χ^2^ tests or, for SNPs with minor allele frequencies <5%, exact tests (Weir, 1996). One SNP (rs12527393 in *E2F3*) had HWE *P-*value <0.001 among controls and was excluded from analysis. Pairwise LD was estimated using *r*^2^ statistics and graphically displayed using the Haploview *v*14.1 (Barrett *et al*, 2005). Unconditional logistic regression analysis was used to estimate OR and 95% CI for risk of ovarian cancer associated with each SNP. Primary tests of association assumed a log-additive (multiplicative) genotypic effect, equivalent to the Armitage test for trend. We also performed separate comparisons of women with one copy (OR_AB *vs* AA_) and two copies (OR_BB *vs* AA_) of the minor allele to women with no copies (reference). Secondary analyses examined dominant and recessive SNP effects. All analyses were adjusted for the design variables of age and geographic region, as well as the following potential confounding variables found to be associated with ovarian cancer risk in the discovery set (*P-*value <0.05): body mass index, postmenopausal hormone use, oral contraceptive use, parity and age at first birth.

Replication association testing was similarly carried out for each SNP using unconditional logistic regression analyses as described above. Associations were examined by site, as well as combined across sites, adjusting for age. Analyses were conducted including and excluding the discovery set participants, adjusting for age and study site. Two sets of *P-*values were calculated for the replication set: one based on the simple comparison-wise error rate and one accounting for the number of replication tests using a Bonferroni correction.

## Results

Distributions of risk factor information for the discovery set have been described earlier (Sellers *et al*, 2005; Kelemen *et al*, 2008). Generally, case–control differences were similar across both discovery sites: overall, cases tended to be more obese, have lower parity, reported a greater family history of ovarian cancer and were more likely to have used hormone therapy (NCO site) or oral contraceptives (MAY site). Of the 288 SNPs attempted, 269 (93.4%) passed quality control and were included in the analysis. Eleven variants in nine genes showing significance at *P-*value <0.05 for adjusted (multivariate) analyses using log-additive (ordinal), recessive or dominant models are shown in Table 2. Assuming a log-additive model, variants in five genes revealed significant associations (*P-*value <0.05): *ABL1* rs2855192, *CDKN1A* rs776246, *CCND3* rs3218086, *CDK2* rs2069414 and *E2F3* rs7760528. SNPs in two of these genes (*ABL1* and *E2F3*) revealed additional evidence of a recessive effect, whereas SNPs in *CDKN1A*, *CCND3* and *CDK2* revealed additional evidence of a dominant association (Table 2). Although our analysis used the log-additive model as the primary analysis, there were two additional SNPs, rs2448343 in *CDC2* and rs12656449 in *CDK7*, with non-significant *P-*values in the log-additive model, but significant *P-*values using a recessive model: OR 0.67 (95% CI 0.50–0.89), *P*=0.006 and OR 2.91 (95% CI 1.11–8.05) *P*=0.03, respectively. *CCNB2* rs1486878 (OR 1.50, 95% CI 1.05–2.15) also suggested association only with a recessive model (*P*=0.04).

**Figure 1:**
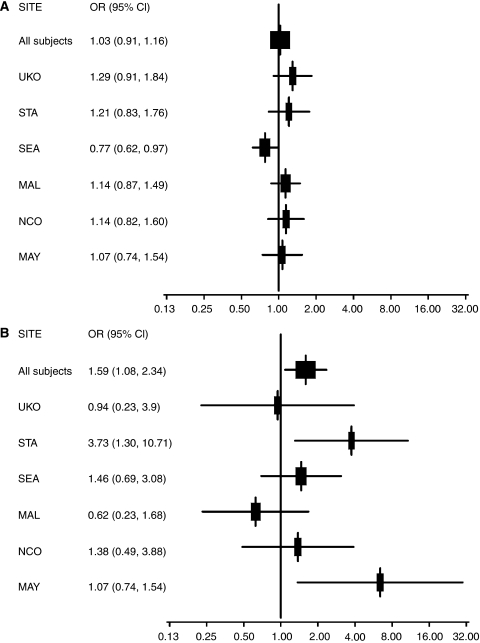
Study-specific and combined OR and 95% CI for *ABL1* rs2855192. Analyses of all subjects adjusted for age and study site; study-specific analyses adjust only for age. (**A**) Heterozygous *vs* homozygous major allele participants (OR_AB *vs* AA_). (**B**) Homozygous minor allele *vs* homozygous major participants (OR_BB *vs* AA_).

Eight of the 11 significant SNPs were chosen for replication. These included *ABL1* rs2855192*, CDKN1A* rs7767246, *CCND3* rs3218086 (which was substituted with rs3218092, *r*^2^−0.95), *E2F3* rs7760528 and *CDK2* rs2069414 (the latter of which was excluded because of lack of TaqMan assay conversion) based on the log-additive model *P-*value < 0.05, *E2F2* rs760607 based on dominant model, *P-*value of 0.02, and *CDC2* rs2448343 and *CDK7* rs12656449 based on recessive model, *P-*value of 0.01 and 0.03, respectively, in the discovery set analysis (Table 2). Table 3 provides results for site-specific and combined replication analyses. For one SNP, rs2855192 in *ABL1*, the results were similar to those obtained in the discovery sample set, in one of the four sites (STA), with a log-additive increase in risk (*P-*value*=*0.03, Table 3; Figure 1) and also consistent with a recessive effect. Combined analysis of all sites revealed a suggestion of a recessive association (OR for homozygous minor allele genotypes compared with homozygous major allele, OR_BB *vs* AA_ 1.59, 95% CI 1.08–2.34, *P-*value=0.02). Excluding the discovery sites, this association was attenuated (OR 1.40, 95% CI 0.89–2.19, *P-*value=0.14) (Table 3). *E2F3* rs776052 was associated with ovarian cancer risk in one replication population (UKO), but did not remain significant in the combined analysis. *CDKN1A* rs776246 and *CDC2* rs2448343 were associated with risk in one population each (MAL and OPS, respectively), but the risk estimates were in the opposite direction to that found in the discovery set and not considered replications. *CDC2* rs2448343 was significantly associated using all datasets assuming a recessive model only. None of the replication results remained statistically significant after correction for multiple testing (data not shown). For SNPs in *CCND3*, *CDK7*, *E2F2* and *E2F3*, no replication of the initial result was seen in any of the replication sites, and the combined analysis did not reveal any significant findings (Table 3; Figure 1).

## Discussion

This study used a two-stage approach to assess the contribution of inherited variation in 39 cell cycle genes to the risk of epithelial ovarian cancer and found some evidence of association at *ABL1* rs2855192. Cell cycle dysregulation is a hallmark of the malignant state, and the function of genetic variation in cell cycle genes, including in ovarian cancer, has been reported in a number of studies (Gayther *et al*, 2007; Goode *et al*, 2009); this study extends the prior findings by the inclusion of an additional 26 and 28 additional genes, respectively. In the discovery set, SNPs in several genes were found to be associated with the risk of ovarian cancer; of these, five genes (*ABL1*, *CCND3*, *CDKN1A*, *E2F3* and *CDK*2) were significant in log-additive models (*P-*value <0.05). This study also found four additional variants in *CCNB2*, *CDC2*, *CDK7* and *E2F2* (rs3328203) to be significant assuming a recessive model only. One additional variant in *E2F2* (rs76067) was found to be associated assuming a dominant model, but not in the log-additive model. Replication testing of seven SNPs revealed one SNP in *ABL1* to have an association in one of the four replication populations assessed (also from the US) and was significant overall with a recessive model. However, once adjustments for multiple comparisons were made, no significant association was noted for any variant.

ABL1 is a ubiquitously expressed, non-tyrosine kinase, encoding both cytoplasmic and nuclear kinases (Preyer *et al*, 2007). The *ABL1* gene is expressed as either a 6 or 7 kb mRNA transcript, with alternatively spliced first exons spliced to exons 2–11. ABL1 has been implicated in processes of cell differentiation, cell division, cell adhesion and cellular stress response (Wang, 1993; Kharbanda *et al*, 1995; Lewis *et al*, 1996; Barila and Superti-Furga, 1998). A t(9;22) translocation, which results in the head-to-tail fusion of the *BCR* and *ABL1* genes, is present in many cases of chronic myelogeneous leukaemia (De Keersmaecker and Cools, 2006). The DNA-binding activity of ABL1 tyrosine kinase is regulated by CDC2-mediated phosphorylation, suggesting a cell cycle function for ABL1 (Welch and Wang, 1993). The tyrosine kinase activity of nuclear ABL1 is regulated in the cell cycle through a specific interaction with Rb (Welch and Wang, 1993). When in the cytoplasm, ABL1 responds to growth factor and adhesion signals to regulate F-actin dynamics (Woodring *et al*, 2003). As acquired resistance to imatinib is associated with mutations in the kinase domain of BCR-ABL that interferes with drug binding, it may be possible that a coding SNP in ABL1 modulates the imatinib response (Crossman *et al*, 2005). The associated SNP, rs2855192, is in intron 1 and the functional aspects are unknown; this SNP was a tagSNP, but did not tag any other SNPs (i.e. it was in a singleton bin with *r*^2^<0.8 with other HapMap SNPs). *ABL1* was included in this study because of its function in cell cycle function; however, the cytoplasmic form of ABL1 may have a function in cell adhesion in addition to DNA binding when localized to the nucleus.

In an earlier study, variants in *CDKN1B* and *CDKNA2/2B* were found to be associated with ovarian cancer risk in a combined analysis of 3601 cases and 5705 controls (Gayther *et al*, 2007). In this study, no variant in either of these genes was significant in the discovery set (Supplementary Table S2) and so were not carried forward to the replication phase. In another study using imputed genotypes, based on data from five independent ovarian cancer studies (Goode *et al*, 2009), the signal observed for *CDNKN1A* in the MAY+NCO dataset was not supported by imputation of genotypes in the other four studies, consistent with the replication data in this report. For rs2069391 in *CDK2* variant, which could not be genotyped in the replication set in this study (discovery set log-additive OR 1.36, CI 1.03–1.78), imputation revealed a signal in the earlier combined analysis (log-additive OR 1.21, CI 1.01–2.09), which included five of the six populations in this study (Goode *et al*, 2009).

A strength of this study was its comprehensive nature in terms of the number of genes and number of tagSNPs and inclusion of putatively functional SNPs. Owing to a large number of tests (269 SNPs × 3 genetic modes of inheritance), caution in interpreting the data is warranted; no adjustment was made for multiple testing because of a lack of complete independence of tests. An additional strength of this study is the inclusion of four replication populations, which improves power (Ioannidis *et al*, 2001; Morgan *et al*, 2007), although replication genotyping of only the top 2% of SNPs limited the power of our two-stage approach. In recent meta-analyses and pooled analyses 161 cancer genetic association studies (Dong *et al*, 2008), close to one-third of all associations were reported to be statistically significant and many of the false positive associations arose from small studies with multiple subset analyses. Therefore, we consider this analysis a preliminary screen of the cell cycle pathway and one which indicates modest evidence for association with disease risk for only one gene, *ABL1*. Additional examination of *ABL1* rs2855192, and including other SNPs with suggestive discovery set results, is warranted in additional studies within the ovarian cancer consortium (Ramus *et al*, 2008).

## Figures and Tables

**Table 1:** Cell cycle related genes

**Gene family**	**Role**	**Genes studied**
Cyclins	Regulate CDK, CDC genes; G1/S, G2/M	*CCNA1, CCNA2, CCNB1, CCNB2, CCND1, CCND2, CCND3, CCNE1, CCNE2, CCNG1, CCNG2*
Cyclin-dependent kinases	Controlled by cylcins and INK4a; late G1/S	*CDK2, CDK4, CDK6, CDK7*
Cell division cycle 2	Regulated by CCNA and CCNB cyclins; G1/S, G2M	*CDC2*
Cell division cycle 25 homologues	Activate CDC2, G1/S	*CDC25A, CDC25B*
Cyclin-dependent kinase inhibitors	Inhibit cyclin–CDK complexes; G1	*CDKN1A, CDKN1B, CDKN2A, CDKN2B, CDKN2C, CDKN2D*
E2F transcription factor family	E2F1, 2, 3 have cyclin-binding domains; most have tumour suppressor, transactivator and dimerization domains; G1/S	*E2F1, E2F2, E2F3, E2F4, E2F5, E2F6*
Transcription factor	Dimerization partners for E2F transcription factors	*TFDP1, TFDP2*
Retinoblastoma-like genes	Like RB, phosphorylated in S and M phases, dephosphorylated in G1, can inhibit transcription of cell cycle genes containing E2F binding sites	*RB1, RBL1, RBL2*
Ubiquitin ligase complex	SCF [SKP1-CUL1-F box] complex, essential element of CDKNA–CDK2 S-phase kinase; SKP2 specifically phosphorylates CDKN1B in S phase	*CUL1, SKP2*
Other	*PLK1* may have a function in localization of CCNB1; *ABL1* DNA binding regulated by CDC2-mediated phosphorylation	*PLK1, ABL1*

**Table 2:** Discovery set: cell cycle SNPs and ovarian cancer risk (*P*<0.05)

	**Samples** **		**Log-additive**	**Heterozygote**	**Homozygote rare allele**	***P*-values**		
**Gene/SNP ID**	**Cases**	**Controls**	**OR (95% CI)**	**OR (95% CI)**	**OR (95% CI)**	**Log-additive**	**Dominant**	**Recessive**
*ABL1*								
rs2855192	825	940	**1.26 (1.03–1.55)**	1.14 (0.90–1.44)	**2.81 (1.29–6.09)**	**0.02**	0.09	**0.01**
								
*CCNB2*								
rs1486878	829	939	1.14 (0.98–1.33)	1.04 (0.85–1.27)	**1.53 (1.06–2.21)**	0.08	0.31	**0.04**
								
*CCND3*								
rs3218086	827	941	**1.23 (1.03–1.47)**	1.18 (0.95–1.47)	**1.75 (1.01–3.03)**	**0.02**	**0.05**	0.07
								
*CDKN1A*								
rs7767246	826	941	**0.83 (0.69–0.99)**	0.83 (0.67–1.02)	0.69 (0.40–1.20)	**0.04**	**0.04**	0.28
								
*CDC2*								
rs2448343	828	935	0.91 (0.79–1.05)	1.13 (0.91–1.39)	0.71 (0.52–0.97)	0.20	0.88	**0.01**
								
*CDK2*								
rs2069414	821	930	**1.36 (1.03–1.78)**	**1.36 (1.02–1.81)**	1.70 (0.27–10.83)	**0.03**	**0.03**	0.60
								
*CDK7*								
Rs12656449	828	941	1.02 (0.8–1.29)	0.86 (0.66–1.13)	**2.91 (1.08–7.86)**	0.90	0.61	**0.03**
								
*E2F2*								
rs3218203	827	941	1.12 (0.95–1.32)	**1.01 (1.08–1.25)**	**1.62 (1.02–2.59)**	0.18	0.47	**0.04**
rs760607	824	941	0.87 (0.75–1.00)	**0.78 (0.63–0.97)**	0.81 (0.60–1.08)	0.05	**0.02**	0.57
								
*E2F3*								
rs7760528	828	940	0.86 (0.75–1.00)	0.93 (0.75–1.14)	**0.69 (0.50–0.97)**	0.05	0.18	**0.04**
rs2328524	829	941	0.88 (0.76–1.01)	0.83 (0.67–1.02)	0.79 (0.59–1.07)	0.06	0.05	0.35

Multivariate logistic hormone replacement therapy, parity and body mass index; *P*-values <0.05 are in bold type, as are CIs that exclude 1.0.

**Table 3:** Discovery and replication sets: cell cycle SNPs and ovarian cancer risk

	**Genotyped samples**		**Log-additive**	**Heterozygote**	**Homozygote rare allele**	***P*-values**		
**Gene/SNP ID**	**Case**	**Control**	**OR (95% CI)**	**OR (95% CI)**	**OR (95% CI)**	**Log-additive**	**Dominant**	**Recessive**
*ABL1 rs2855192*								
MAL	431	1199	1.04 (0.82–1.32)	1.14 (0.87–1.49)	0.62 (0.23–1.68)	0.75	0.51	0.32
SEA	693	1219	0.86 (0.71–1.05)	**0.77 (0.62–0.97)**	1.46 (0.69–3.08)	0.14	**0.05**	0.24
STA	284	364	**1.42 (1.05–1.93)**	1.21 (0.83–1.76)	**3.73 (1.30–10.7)**	0.03	0.09	**0.02**
UKO	265	579	1.23 (0.89–1.69)	1.29 (0.91–1.84)	0.94 (0.23–3.91)	0.21	0.17	0.86
Combined replication	1673	3361	1.04 (0.92–1.18)	1.00 (0.87–1.15)	1.40 (0.89–2.19)	0.48	0.73	0.14
Combined all	2322	4269	1.08 (0.97–1.21)	1.03 (0.91–1.16)	**1.59 (1.08–2.34)**	0.14	0.35	**0.02**
								
*CCND3 rs3218086*								
MAL	424	1186	1.14 (0.94–1.39)	1.21 (0.95–1.54)	1.08 (0.61–1.93)	0.19	0.12	0.96
SEA	594	849	0.96 (0.78–1.17)	1.02 (0.81–1.29)	0.69 (0.35–1.35)	0.67	0.92	0.26
STA	283	363	0.84 (0.64–1.10)	0.92 (0.65–1.31)	0.56 (0.26–1.22)	0.21	0.38	0.16
UKO	–	–				–	–	
Combined replication	1301	2398	1.00 (0.89–1.14)	1.07 (0.92–1.25)	0.81 (0.55–1.91)	0.95	0.59	0.23
Combined all	1951	3307	1.09 (0.98–1.21)	1.12 (0.99–1.27)	1.07 (0.79–1.46)	0.10	0.07	0.83
								
*CDC2 rs2448343*								
MAL	419	1159	1.02 (0.87–1.20)	1.08 (0.85–1.38)	0.99 (0.69–1.41)	0.83	0.61	0.77
SEA	691	1212	0.95 (0.82–1.09)	0.99 (0.81–1.21)	0.87 (0.64–1.17)	0.45	0.68	0.35
STA	279	359	1.14 (0.90–1.44)	1.12 (0.80–1.57)	1.31 (0.78–2.20)	0.28	0.37	0.40
UKO	252	571	0.80 (0.63–1.01)	**0.69 (0.49–0.98)**	0.71 (0.43–1.16)	0.06	**0.03**	0.50
Combined replication	1640	3301	0.97 (0.89–1.06)	0.99 (0.87–1.13)	0.92 (0.76–1.10)	0.48	0.70	0.39
Combined all	2290	4204	0.96 (0.89–1.04)	1.05 (0.94–1.17)	0.87 (0.73–1.03)	0.32	0.96	0.04
								
*CDK7 rs12656449*								
MAL	434	1205	0.98 (0.75–1.29)	1.01 (0.75–1.35)	0.75 (0.21–2.74)	0.90	0.97	0.66
SEA	692	1220	1.06 (0.84–1.33)	1.12 (0.88–1.45)	0.57 (0.18–1.83)	0.64	0.46	0.33
STA	283	361	0.83 (0.55–1.27)	0.83 (0.52–1.34)	0.70 (0.11–4.23)	0.40	0.41	0.71
UKO	258	470	1.17 (0.78–1.75)	1.16 (0.76–1.77)	1.64 (0.15–18.2)	0.45	0.47	0.70
Combined replication	1667	3356	1.01 (0.87–1.17)	1.05 (0.89–1.23)	0.69 (0.33–1.44)	0.89	0.70	0.31
Combined all	2318	4265	1.01 (0.89–1.14)	0.99 (0.86–1.14)	1.19 (0.69–2.05)	0.90	0.98	0.53
								
*CDKN1A rs776246*								
MAL	428	1202	1.21 (1.00–1.46)	**1.28 (1.01–1.63)**	1.26 (0.73–2.16)	0.05	**0.04**	0.59
SEA	691	1216	0.97 (0.82–1.14)	1.02 (0.83–1.25)	0.78 (0.47–1.30)	0.68	0.94	0.33
STA	284	355	1.17 (0.89–1.55)	1.18 (0.84–1.65)	1.37 (0.59–3.13)	0.26	0.28	0.54
UKO	262	575	0.97 (0.73–1.28)	1.10 (0.78–1.55)	0.61 (0.24–1.54)	0.81	0.86	0.26
Combined replication	1665	3348	1.08 (0.97–1.20)	1.14 (1.00–1.30)	0.98 (0.72–1.34)	0.15	0.07	0.71
Combined all	2314	4257	1.01 (0.92–1.10)	1.05 (0.94–1.18)	0.88 (0.67–1.16)	0.86	0.54	0.31
								
*E2F2 rs760607*								
MAL	421	1159	0.91 (0.77–1.07)	0.89 (0.70–1.14)	0.83 (0.58–1.20)	0.25	0.27	0.48
SEA	692	1221	0.99 (0.86–1.13)	0.99 (0.80–1.21)	0.98 (0.73–1.31)	0.87	0.87	0.92
STA	281	346	1.21 (0.97–1.52)	1.19 (0.83–1.69)	1.48 (0.93–2.35)	0.09	0.17	0.16
UKO	261	574	1.12 (0.89–1.40)	0.88 (0.62–1.25)	1.40 (0.89–2.20)	0.33	0.99	0.06
Combined replication	1655	3300	1.01 (0.93–1.10)	0.94(0.83–1.08)	1.06 (0.88–1.27)	0.80	0.70	0.30
Combined all	2304	4209	0.98 (0.91–1.05)	0.91 (0.81–1.02)	1.00 (0.86–1.17)	0.58	0.19	0.48
								
*E2F3 rs7760528*								
MAL	426	1200	1.04 (0.89–1.24)	1.08 (0.85–1.36)	1.07 (0.73–1.56)	0.58	0.53	0.86
SEA	692	1215	0.94 (0.81–1.08)	1.01 (0.83–1.24)	0.80 (0.57–1.11)	0.36	0.74	0.15
STA	282	361	0.99 (0.79–1.25)	1.03 (0.74–1.44)	0.97 (0.58–1.61)	0.99	0.91	0.84
UKO	259	576	1.24 (0.97–1.57)	1.01 (0.73–1.43)	**1.85 (1.10–3.11)**	0.08	0.38	**0.02**
Combined replication	1659	3352	1.01 (0.93–1.11)	1.04 (0.91–1.17)	1.00 (0.82–1.23)	0.77	0.64	0.90
Combined all	2309	4260	0.96 (0.89–1.04)	0.99 (0.89–1.11)	0.89 (0.75–1.07)	0.34	0.62	0.21

Logistic regression analysis adjusting for age and, for combined results, study site; *P*-values ⩽0.05 and CIs that exclude 1.0 are in bold type; combined all indicates discovery plus replication sets; *CCND3* rs32189092 was substituted for *CCND3* rs3218086 in replication sets.
