# Supplementary material for: Cell cycle genes and ovarian cancer susceptibility: a tagSNP analysis
Source: Br J Cancer. 2009 Sep 8;101(8):1461–8. doi: 10.1038/sj.bjc.6605284 (PMC2768434; doi:10.1038/sj.bjc.6605284)
Supplement: Supplementary Table S1–S2 [file 6605284x1.doc]

**Supplemental Table 1. Cell Cycle Gene Information**

| **Gene Name** | **GeneID** | **RefSeq** | **Chr** | **Str** | | **Start (bp)** | | **Size (kb)** | **Description** | **Tag** | **Func** | **Other** | **N SNPs (N Passed QC)** | |
| --- | --- | --- | --- | --- | --- | --- | --- | --- | --- | --- | --- | --- | --- | --- |
| *E2F2* | 1870 | NM_004091.2 | 1 | | - | | 23,730,300 | 24.8 | E2F transcription factor 2 | HapMap | p-f | - | | 9 (9) |
| *CDKN2C* | 1031 | NM_001262.2 | 1 | | + | | 51,206,955 | 5.9 | Cyclin-dependent kinase inhibitor 2C (p18, inhibits CDK4) | HapMap | p-f | - | | 1 (1) |
| *E2F6* | 1876 | NM_198256.2 | 2 | | - | | 11,523,748 | 21.8 | E2F transcription factor 6 | HapMap | p-f | - | | 4 (4) |
| *CDC25A* | 993 | NM_001789.2 | 3 | | - | | 48,204,805 | 31.1 | Cell division cycle 25 homolog A (S. pombe) | HapMap | p-f | - | | 2 (2) |
| *TFDP2* | 7029 | NM_006286.3 | 3 | | - | | 143,207,067 | 53.0 | Transcription factor Dp-2 (E2F dimerization partner 2) | HapMap | p-f | - | | 10 (9) |
| *CCNG2* | 901 | NM_004354.1 | 4 | | + | | 78,297,551 | 9.4 | Cyclin G2 | HapMap | p-f | - | | 4 (4) |
| *CCNA2* | 890 | NM_001237.2 | 4 | | - | | 122,964,342 | 6.4 | Cyclin A2 | Perlegen | p-f | - | | 3 (3) |
| *SKP2* | 6502 | NM_005983.2 | 5 | | + | | 36,187,946 | 30.1 | S-phase kinase-associated protein 2 (p45) | HapMap | p-f | - | | 14 (14) |
| *CCNB1* | 891 | NM_031966.2 | 5 | | + | | 68,498,669 | 11.2 | Cyclin B1 | HapMap | p-f | - | | 6 (6) |
| *CDK7* | 1022 | NM_001799.2 | 5 | | + | | 68,566,471 | 42.5 | Cyclin-dependent kinase 7 | HapMap | p-f | - | | 3 (3) |
| *CCNG1* | 900 | NM_004060.3 | 5 | | + | | 162,797,155 | 7.4 | Cyclin G1 | Perlegen | p-f | - | | 4 (4) |
| *E2F3* | 1871 | NM_001949.2 | 6 | | + | | 20,510,377 | 91.5 | E2F transcription factor 3 | - | - | HapMap-m | | 16 (16) |
| *CDKN1A* | 1026 | NM_078467.1 | 6 | | + | | 36,754,465 | 8.6 | Cyclin-dependent kinase inhibitor 1A (p21, Cip1) | HapMap | p-f | - | | 4 (4) |
| *CCND3* | 896 | NM_001760.2 | 6 | | - | | 42,017,530 | 6.9 | Cyclin D3 | HapMap | p-f | - | | 7 (7) |
| *CDK6* | 1021 | NM_001259.5 | 7 | | - | | 92,301,148 | 229.0 | Cyclin-dependent kinase 6 | HapMap | p-f | - | | 39 (33) |
| *CUL1* | 8454 | NM_003592.2 | 7 | | + | | 148,026,866 | 102.3 | Cullin 1 | - | - | HapMap-m | | 37 (33) |
| *E2F5* | 1875 | NM_001951.3 | 8 | | + | | 86,276,871 | 37.1 | E2F transcription factor 5, p130-binding | HapMap | p-f | - | | 6 (5) |
| *CCNE2* | 9134 | NM_057749.1 | 8 | | - | | 95,976,660 | 15.0 | Cyclin E2 | HapMap | p-f | - | | 7 (7) |
| *CDKN2A* | 1029 | NM_058195.2 | 9 | | - | | 21,965,038 | 7.3 | Cyclin-dependent kinase inhibitor 2A (melanoma, p16, inhibits CDK4) | HapMap | p-f | - | | 3 (3) |
| *CDKN2B* | 1030 | NM_004936.3 | 9 | | - | | 21,999,312 | 6.4 | Cyclin-dependent kinase inhibitor 2B (p15, inhibits CDK4) | HapMap | p-f | - | | 6 (6) |
| *ABL1* | 25 | NM_007313.2 | 9 | | + | | 132,700,652 | 52.2 | C-abl oncogene 1, receptor tyrosine kinase | HapMap | p-f | - | | 19 (17) |
| *CDC2* | 983 | NM_001786.2 | 10 | | + | | 62,205,690 | 18.2 | Cell division cycle 2, G1 to S and G2 to M | HapMap | p-f | - | | 11 (10) |
| *CCND1* | 595 | NM_053056.2 | 11 | | + | | 69,165,054 | 13.4 | Cyclin D1 | HapMap | p-f | - | | 5 (5) |
| *CCND2* | 894 | NM_001759.2 | 12 | | + | | 4,253,199 | 31.6 | Cyclin D2 | - | p-f | - | | 4 (4) |
| *CDKN1B* | 1027 | NM_004064.2 | 12 | | + | | 12,761,576 | 5.0 | Cyclin-dependent kinase inhibitor 1B (p27, Kip1) | HapMap | p-f | - | | 8 (8) |
| *CDK2* | 1017 | NM_001798.2 | 12 | | + | | 54,646,826 | 6.0 | Cyclin-dependent kinase 2 | HapMap | p-f | - | | 7 (7) |
| *CDK4* | 1019 | NM_000075.2 | 12 | | - | | 56,432,431 | 4.2 | Cyclin-dependent kinase 4 | HapMap | p-f | - | | 3 (3) |
| *CCNA1* | 8900 | NM_003914.2 | 13 | | + | | 35,904,633 | 10.4 | Cyclin A1 | HapMap | p-f | - | | 5 (5) |
| *RB1* | 5925 | NM_000321.2 | 13 | | + | | 47,775,884 | 178.1 | Retinoblastoma 1 (including osteosarcoma) | - | p-f | - | | 8 (8) |
| *TFDP1* | 7027 | NM_007111.3 | 13 | | + | | 113,287,057 | 56.4 | Transcription factor Dp-1 | HapMap | p-f | - | | 9 (7) |
| *CCNB2* | 9133 | NM_004701.2 | 15 | | + | | 57,184,612 | 19.9 | Cyclin B2 | HapMap | p-f | - | | 3 (3) |
| *PLK1* | 5347 | NM_005030.3 | 16 | | + | | 23,597,702 | 11.5 | Polo-like kinase 1 (Drosophila) | - | p-f | - | | 1 (1) |
| *RBL2* | 5934 | NM_005611.2 | 16 | | + | | 52,025,901 | 57.2 | Retinoblastoma-like 2 (p130) | Perlegen | p-f | - | | 6 (6) |
| *E2F4* | 1874 | NM_001950.3 | 16 | | + | | 65,783,569 | 6.8 | E2F transcription factor 4, p107/p130-binding | HapMap | p-f | - | | 1 (1) |
| *CDKN2D* | 1032 | NM_001800.3 | 19 | | - | | 10,540,631 | 2.5 | Cyclin-dependent kinase inhibitor 2D (p19, inhibits CDK4) | HapMap | p-f | - | | 2 (2) |
| *CCNE1* | 898 | NM_001238.1 | 19 | | + | | 34,994,741 | 12.3 | Cyclin E1 | HapMap | p-f | - | | 2 (2) |
| *CDC25B* | 994 | NM_021873.2 | 20 | | + | | 3,724,401 | 10.4 | Cell division cycle 25 homolog B (S. pombe) | - | - | HapMap-m | | 1 (1) |
| *E2F1* | 1869 | NM_005225.1 | 20 | | - | | 31,737,854 | 10.7 | E2F transcription factor 1 | HapMap | p-f | - | | 3 (2) |
| *RBL1* | 5933 | NM_002895.2 | 20 | | - | | 35,157,824 | 98.2 | Retinoblastoma-like 1 (p107) | HapMap | p-f | - | | 5 (4) |
| Genome build 36.3; Refseq Release 29 (May 4, 2008); chr, chromosome, str, strand; Functional SNPs indicates that 5’ UTR, 3’ UTR, non-synonymous, and 1 kb from start SNPs were included if MAF > 0.05 and Illumina-provide SNP_Score > 0.6; Tag, Func, Other indicate SNP selection approach used, with the source listed in Tag as the most informative from the four sources examined, additional detail is provided in the text; p-f indicates SNPs within 1 kb upstream, 5′ UTR, 3′ UTR, or non-synonymous with MAF ≥ 0.05 were additionally included; HapMap-m indicates singleton bins were excluded; N analyzed SNPs excludes 10 (3.5%) which failed genotyping and 8 (2.8%) with incorrect genomic coordinates or low MAF. | | | | | | | | | | | | | | |

**Supplemental Table S2. Quality Metrics, Odds Ratios (OR), and 95% Confidence Intervals (CI)**

| **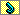Gene** | **SNP rsid** | **Chr** | **Position (bp)** | **Location** | **Alleles** | **Call Rate** | **MAF,**  **Controls** | **HWE**  **p-value** | **OR (95% CI),**  **Heterozygotes** | **OR (95% CI),**  **Homozygous Rare Allele** | **log-additive**  **p-value** |
| --- | --- | --- | --- | --- | --- | --- | --- | --- | --- | --- | --- |
| *E2F2* | rs3820028 | 1 | 23,707,073 | 3' UTR | G>A | 1.00 | 0.51 | 0.17 | 0.78 (0.61-0.99) | 0.87 (0.66-1.14) | 0.32 |
|  | rs3218211 | 1 | 23,708,381 | 3' UTR | A>G | 1.00 | 0.51 | 0.18 | 0.78 (0.61-0.99) | 0.88 (0.67-1.15) | 0.37 |
|  | rs2075993 | 1 | 23,708,951 | 3' UTR | A>G | 1.00 | 0.51 | 0.18 | 0.77 (0.61-0.98) | 0.87 (0.66-1.14) | 0.35 |
|  | rs3218203 | 1 | 23,710,147 | intron | C>G | 1.00 | 0.20 | 0.57 | 1.01 (0.82-1.25) | 1.62 (1.02-2.59) | 0.18 |
|  | rs2742976 | 1 | 23,730,589 | 5' (289) | C>A | 1.00 | 0.37 | 0.66 | 0.91 (0.74-1.12) | 1.06 (0.78-1.42) | 0.96 |
|  | rs3218121 | 1 | 23,730,904 | 5' (604) | G>A | 1.00 | 0.08 | 0.85 | 0.78 (0.59-1.04) | 0.70 (0.20-2.41) | 0.07 |
|  | rs6667575 | 1 | 23,732,108 | 5' (1,808) | G>A | 1.00 | 0.26 | 0.39 | 0.90 (0.73-1.10) | 0.91 (0.60-1.36) | 0.32 |
|  | rs763586 | 1 | 23,732,620 | 5' (2,320) | C>T | 1.00 | 0.08 | 0.82 | 0.79 (0.59-1.05) | 0.70 (0.20-2.42) | 0.09 |
|  | rs760607 | 1 | 23,735,240 | 5' (4,940) | T>C | 1.00 | 0.40 | 0.37 | 0.78 (0.63-0.97) | 0.81 (0.60-1.08) | 0.05 |
| *CDKN2C* | rs12855 | 1 | 51,212,681 | 3' UTR | C>T | 1.00 | 0.09 | 0.23 | 1.09 (0.84-1.42) | 0.43 (0.13-1.38) | 0.99 |
| *E2F6* | rs13414905 | 2 | 11,498,020 | 3' (3,932) | T>G | 1.00 | 0.24 | 0.02 | 0.95 (0.78-1.16) | 1.44 (0.93-2.22) | 0.50 |
|  | rs6432193 | 2 | 11,501,448 | 3' (504) | C>G | 1.00 | 0.37 | 0.15 | 0.98 (0.79-1.21) | 0.99 (0.73-1.35) | 0.89 |
|  | rs6709507 | 2 | 11,518,655 | intron | C>T | 1.00 | 0.05 | 0.43 | 1.06 (0.77-1.47) | 0.96 (0.20-4.56) | 0.74 |
|  | rs10192676 | 2 | 11,522,431 | intron | C>T | 1.00 | 0.40 | 0.37 | 1.03 (0.84-1.28) | 0.93 (0.69-1.24) | 0.76 |
| *CDC25A* | rs6797587 | 3 | 48,172,618 | 3' (1,054) | C>T | 1.00 | 0.32 | 0.72 | 1.03 (0.84-1.26) | 1.19 (0.86-1.64) | 0.36 |
|  | rs936426 | 3 | 48,190,257 | intron | G>A | 1.00 | 0.30 | 0.96 | 1.02 (0.83-1.25) | 1.17 (0.84-1.64) | 0.45 |
| *TFDP2* | rs2163294 | 3 | 143,151,143 | 3' (2,875) | C>A | 1.00 | 0.37 | 0.16 | 1.22 (0.99-1.51) | 1.16 (0.87-1.55) | 0.16 |
|  | rs3804771 | 3 | 143,174,097 | intron | C>T | 1.00 | 0.15 | 0.16 | 1.21 (0.97-1.51) | 1.21 (0.70-2.11) | 0.09 |
|  | rs7642874 | 3 | 143,180,562 | intron | C>T | 1.00 | 0.31 | 0.22 | 1.11 (0.90-1.36) | 0.92 (0.65-1.29) | 0.88 |
|  | rs12493472 | 3 | 143,190,784 | intron | G>C | 1.00 | 0.11 | 0.49 | 0.99 (0.78-1.27) | 0.64 (0.22-1.87) | 0.72 |
|  | rs3811708 | 3 | 143,190,861 | intron | G>T | 1.00 | 0.14 | 0.12 | 1.05 (0.84-1.32) | 1.03 (0.57-1.86) | 0.71 |
|  | rs6440055 | 3 | 143,191,773 | intron | C>T | 1.00 | 0.15 | 0.52 | 0.99 (0.79-1.24) | 1.11 (0.61-2.01) | 0.91 |
|  | rs733996 | 3 | 143,222,766 | 5' (15,699) | C>T | 1.00 | 0.05 | 0.85 | 0.98 (0.71-1.35) | 0.86 (0.14-5.34) | 0.86 |
|  | rs7644383 | 3 | 143,226,641 | 5' (19,574) | A>G | 1.00 | 0.25 | 0.24 | 1.13 (0.93-1.39) | 0.92 (0.62-1.37) | 0.62 |
|  | rs963396 | 3 | 143,233,229 | 5' (26,162) | A>G | Failed | - | - | - | - | - |
|  | rs11720300 | 3 | 143,248,346 | 5' (41,279) | T>A | 1.00 | 0.08 | 0.38 | 1.01 (0.77-1.32) | 1.53 (0.41-5.70) | 0.78 |
| *CCNG2* | rs6849534 | 4 | 78,292,679 | 5' (4,872) | G>T | 1.00 | 0.5 | 0.82 | 0.89 (0.70-1.12) | 0.96 (0.73-1.26) | 0.78 |
|  | rs10010725 | 4 | 78,294,646 | 5' (2,905) | T>C | 1.00 | 0.08 | 0.09 | 1.16 (0.88-1.53) | 0.61 (0.21-1.73) | 0.63 |
|  | rs4544732 | 4 | 78,294,858 | 5' (2,693) | G>A | 1.00 | 0.43 | 0.19 | 0.93 (0.75-1.16) | 1.01 (0.76-1.34) | 0.90 |
|  | rs4150084 | 4 | 78,303,928 | intron | G>A | 1.00 | 0.08 | 0.15 | 1.17 (0.89-1.52) | 0.81 (0.29-2.31) | 0.41 |
| *CCNA2* | rs3217772 | 4 | 122,958,633 | intron | C>G | 1.00 | 0.39 | 0.53 | 1.09 (0.88-1.35) | 1.03 (0.77-1.38) | 0.67 |
|  | rs2706793 | 4 | 122,968,886 | 5' (4,544) | A>C | 1.00 | 0.27 | 0.43 | 1.11 (0.90-1.36) | 0.98 (0.67-1.43) | 0.60 |
|  | rs1507994 | 4 | 122,968,991 | 5' (4,649) | C>T | 0.99 | 0.32 | 0.03 | 1.04 (0.85-1.27) | 0.75 (0.51-1.11) | 0.49 |
| *SKP2* | rs10071838 | 5 | 36,183,563 | 5' (4,383) | G>A | 1.00 | 0.38 | 0.04 | 0.79 (0.64-0.97) | 0.96 (0.71-1.30) | 0.28 |
|  | rs33670 | 5 | 36,185,177 | 5' (2,769) | A>C | 1.00 | 0.13 | 0.13 | 0.98 (0.78-1.23) | 1.87 (0.83-4.21) | 0.59 |
|  | rs33671 | 5 | 36,188,499 | intron | G>A | 1.00 | 0.12 | 0.49 | 1.01 (0.80-1.27) | 1.40 (0.64-3.08) | 0.65 |
|  | rs12655652 | 5 | 36,189,803 | intron | C>T | 1.00 | 0.40 | 0.43 | 0.89 (0.72-1.10) | 1.08 (0.81-1.44) | 0.92 |
|  | rs3804446 | 5 | 36,192,539 | intron | A>G | 1.00 | 0.13 | 0.84 | 1.06 (0.84-1.33) | 1.05 (0.50-2.18) | 0.66 |
|  | rs33678 | 5 | 36,193,572 | intron | C>T | 1.00 | 0.11 | 0.62 | 1.11 (0.87-1.41) | 1.27 (0.58-2.78) | 0.31 |
|  | rs12656216 | 5 | 36,196,425 | intron | T>C | 1.00 | 0.22 | 0.59 | 1.05 (0.85-1.29) | 1.05 (0.68-1.64) | 0.65 |
|  | rs155543 | 5 | 36,196,964 | intron | G>T | 1.00 | 0.06 | 0.30 | 0.84 (0.62-1.13) | 2.90 (0.55-15.4) | 0.53 |
|  | rs7715070 | 5 | 36,207,690 | intron | A>C | 1.00 | 0.05 | 0.49 | 1.00 (0.72-1.39) | 1.37 (0.27-6.89) | 0.89 |
|  | rs3804439 | 5 | 36,214,801 | intron | T>C | 1.00 | 0.11 | 0.48 | 1.04 (0.82-1.33) | 0.75 (0.32-1.78) | 0.98 |
|  | rs3804437 | 5 | 36,216,179 | intron | T>C | 1.00 | 0.06 | 0.67 | 0.95 (0.70-1.28) | 1.26 (0.25-6.38) | 0.80 |
|  | rs17279275 | 5 | 36,216,267 | intron | A>G | 1.00 | 0.27 | 0.23 | 0.88 (0.72-1.07) | 1.07 (0.73-1.59) | 0.58 |
|  | rs27130 | 5 | 36,216,544 | intron | C>T | 1.00 | 0.17 | 0.20 | 1.07 (0.86-1.34) | 1.05 (0.62-1.79) | 0.56 |
|  | rs7731023 | 5 | 36,217,384 | intron | C>T | 1.00 | 0.44 | 0.13 | 0.86 (0.69-1.07) | 1.05 (0.79-1.39) | 0.98 |
| *CCNB1* | rs352626 | 5 | 68,497,959 | 5' (710) | C>T | 1.00 | 0.41 | 0.14 | 1.08 (0.87-1.34) | 0.94 (0.71-1.25) | 0.85 |
|  | rs350104 | 5 | 68,498,194 | 5' (475) | A>G | 1.00 | 0.48 | 0.22 | 1.14 (0.90-1.43) | 0.99 (0.75-1.30) | 1.00 |
|  | rs164390 | 5 | 68,498,770 | 5' UTR | G>T | 1.00 | 0.41 | 0.08 | 1.09 (0.88-1.35) | 0.92 (0.70-1.22) | 0.78 |
|  | rs163444 | 5 | 68,500,300 | intron | G>A | 1.00 | 0.48 | 0.30 | 1.14 (0.90-1.43) | 0.97 (0.74-1.27) | 0.9 |
|  | rs2069432 | 5 | 68,503,168 | intron | C>T | 1.00 | 0.11 | 0.06 | 1.03 (0.81-1.30) | 1.83 (0.64-5.21) | 0.53 |
|  | rs2069433 | 5 | 68,503,263 | intron | A>G | 1.00 | 0.08 | 0.87 | 0.85 (0.64-1.12) | 2.64 (0.96-7.29) | 0.92 |
| *CDK7* | rs2972381 | 5 | 68,573,998 | intron | G>A | 1.00 | 0.47 | 0.18 | 1.12 (0.89-1.40) | 0.92 (0.70-1.20) | 0.63 |
|  | rs4423955 | 5 | 68,603,731 | intron | C>T | 1.00 | 0.33 | 0.18 | 1.15 (0.94-1.41) | 0.86 (0.62-1.19) | 0.95 |
|  | rs12656449 | 5 | 68,605,243 | intron | G>A | 1.00 | 0.09 | 0.62 | 0.86 (0.66-1.13) | 2.91 (1.08-7.86) | 0.90 |
| *CCNG1* | rs2069347 | 5 | 162,799,773 | intron | A>G | 1.00 | 0.48 | 0.47 | 0.98 (0.78-1.23) | 1.05 (0.80-1.38) | 0.76 |
|  | rs6889776 | 5 | 162,805,591 | 3' (991) | T>A | 1.00 | 0.05 | 1.00 | 0.94 (0.67-1.31) | 1.15 (0.16-8.50) | 0.75 |
|  | rs422356 | 5 | 162,806,085 | 3' (1,485) | T>C | 1.00 | 0.31 | 0.97 | 0.95 (0.78-1.17) | 0.88 (0.62-1.24) | 0.44 |
|  | rs177250 | 5 | 162,807,979 | 3' (3,379) | T>C | 1.00 | 0.26 | 0.34 | 1.01 (0.82-1.24) | 0.82 (0.56-1.21) | 0.55 |
| *E2F3* | rs9465733 | 6 | 20,517,754 | intron | C>A | 1.00 | 0.35 | 0.81 | 0.99 (0.80-1.22) | 1.13 (0.83-1.54) | 0.57 |
|  | rs12527393 | 6 | 20,522,319 | intron | A>G | 1.00 | 0.27 | <.01 | 0.83 (0.68-1.02) | 1.17 (0.77-1.79) | 0.46 |
|  | rs17569598 | 6 | 20,522,777 | intron | C>T | 0.99 | 0.37 | 0.85 | 0.99 (0.81-1.23) | 0.97 (0.71-1.31) | 0.84 |
|  | rs911361 | 6 | 20,523,032 | intron | C>T | 1.00 | 0.42 | 0.48 | 0.96 (0.77-1.20) | 1.22 (0.92-1.61) | 0.24 |
|  | rs2328489 | 6 | 20,526,382 | intron | G>C | 1.00 | 0.28 | 0.61 | 0.85 (0.69-1.04) | 0.83 (0.57-1.22) | 0.11 |
|  | rs7752992 | 6 | 20,533,437 | intron | C>T | 1.00 | 0.40 | 0.79 | 0.95 (0.77-1.17) | 0.87 (0.65-1.17) | 0.37 |
|  | rs9465741 | 6 | 20,536,411 | intron | A>C | 1.00 | 0.48 | 0.88 | 0.98 (0.78-1.24) | 0.80 (0.61-1.06) | 0.14 |
|  | rs7760528 | 6 | 20,550,333 | intron | T>C | 1.00 | 0.34 | 0.46 | 0.93 (0.75-1.14) | 0.69 (0.50-0.97) | 0.05 |
|  | rs6926668 | 6 | 20,575,557 | intron | A>G | 1.00 | 0.22 | 0.96 | 0.86 (0.69-1.06) | 1.06 (0.68-1.66) | 0.41 |
|  | rs4134938 | 6 | 20,589,657 | intron | C>G | 1.00 | 0.22 | 0.35 | 0.91 (0.74-1.12) | 1.05 (0.66-1.69) | 0.60 |
|  | rs4134939 | 6 | 20,589,804 | intron | C>T | 1.00 | 0.29 | 0.92 | 1.09 (0.89-1.33) | 1.16 (0.81-1.66) | 0.31 |
|  | rs3806114 | 6 | 20,590,314 | intron | A>G | 1.00 | 0.32 | 0.22 | 1.05 (0.86-1.29) | 1.17 (0.83-1.65) | 0.36 |
|  | rs7747931 | 6 | 20,590,390 | intron | C>T | 1.00 | 0.45 | 0.41 | 1.09 (0.87-1.37) | 1.05 (0.79-1.39) | 0.67 |
|  | rs11964747 | 6 | 20,593,877 | intron | G>A | 1.00 | 0.18 | 0.80 | 0.91 (0.73-1.13) | 1.21 (0.71-2.07) | 0.81 |
|  | rs3806116 | 6 | 20,595,229 | intron | C>A | 1.00 | 0.37 | 0.87 | 1.14 (0.92-1.41) | 1.25 (0.93-1.68) | 0.11 |
|  | rs2328524 | 6 | 20,596,213 | intron | C>T | 1.00 | 0.40 | 0.75 | 0.83 (0.67-1.02) | 0.79 (0.59-1.07) | 0.06 |
| *CDKN1A* | rs1977172 | 6 | 36,749,732 | 5' (4,733) | C>A | 1.00 | 0.12 | 0.92 | 1.14 (0.91-1.44) | 1.12 (0.52-2.43) | 0.27 |
|  | rs3829963 | 6 | 36,752,364 | 5' (2,101) | G>T | 1.00 | 0.13 | 0.25 | 1.15 (0.91-1.45) | 1.01 (0.50-2.03) | 0.33 |
|  | rs733590 | 6 | 36,753,181 | 5' (1,284) | T>C | 0.99 | 0.38 | 0.14 | 0.90 (0.73-1.11) | 0.81 (0.59-1.11) | 0.15 |
|  | rs7767246 | 6 | 36,767,193 | 3' (4,106) | C>G | 1.00 | 0.21 | 0.20 | 0.83 (0.67-1.02) | 0.69 (0.40-1.20) | 0.04 |
| *CCND3* | rs2479726 | 6 | 42,006,074 | 3' (4,575) | G>A | 0.99 | 0.29 | 0.57 | 0.92 (0.75-1.13) | 0.93 (0.65-1.31) | 0.47 |
|  | rs3828855 | 6 | 42,008,384 | 3' (2,265) | G>A | 1.00 | 0.10 | 0.03 | 0.91 (0.71-1.17) | 1.45 (0.30-6.93) | 0.56 |
|  | rs3218108 | 6 | 42,010,633 | 3' (16) | G>A | 1.00 | 0.27 | 0.42 | 0.92 (0.75-1.13) | 0.99 (0.67-1.47) | 0.60 |
|  | rs9529 | 6 | 42,010,985 | 3' UTR | C>T | 1.00 | 0.29 | 0.87 | 0.93 (0.76-1.14) | 0.89 (0.62-1.28) | 0.42 |
|  | rs3218086 | 6 | 42,018,042 | 5' (512) | C>T | 1.00 | 0.16 | 0.89 | 1.18 (0.95-1.47) | 1.75 (1.01-3.03) | 0.02 |
|  | rs3218085 | 6 | 42,018,157 | 5' (627) | G>T | 1.00 | 0.01 | 1.00 | 1.15 (0.63-2.10) | - | 0.65 |
|  | rs9381100 | 6 | 42,019,163 | 5' (1,633) | A>G | 1.00 | 0.24 | 0.58 | 0.91 (0.74-1.12) | 0.71 (0.46-1.10) | 0.12 |
| *CDK6* | rs6972637 | 7 | 91,444,880 | 3' (627,291) | A>G | Incorrect tagging | - | - | - | - | - |
|  | rs10488509 | 7 | 91,455,027 | 3' (617,144) | C>G | Incorrect tagging | - | - | - | - | - |
|  | rs1989779 | 7 | 91,470,242 | 3' (601,929) | A>G | Incorrect tagging | - | - | - | - | - |
|  | rs2158138 | 7 | 91,538,815 | 3' (533,356) | C>T | Incorrect tagging | - | - | - | - | - |
|  | rs2049900 | 7 | 91,576,724 | 3' (495,447) | G>C | Incorrect tagging | - | - | - | - | - |
|  | rs6465353 | 7 | 91,617,907 | 3' (454,264) | T>G | Incorrect tagging | - | - | - | - | - |
|  | rs8179 | 7 | 92,074,100 | 3' UTR | G>A | 1.00 | 0.20 | 0.19 | 1.20 (0.97-1.48) | 1.22 (0.77-1.91) | 0.09 |
|  | rs42030 | 7 | 92,074,357 | 3' UTR | C>T | 1.00 | 0.07 | <0.01 | 1.42 (1.07-1.90) | 0.38 (0.12-1.23) | 0.19 |
|  | rs4272 | 7 | 92,074,765 | 3' UTR | T>C | 1.00 | 0.21 | 0.29 | 1.15 (0.93-1.42) | 1.24 (0.80-1.94) | 0.13 |
|  | rs42031 | 7 | 92,075,332 | 3' UTR | A>T | 1.00 | 0.20 | 0.19 | 1.20 (0.97-1.48) | 1.25 (0.80-1.96) | 0.08 |
|  | rs42034 | 7 | 92,077,080 | 3' UTR | A>G | 1.00 | 0.25 | 0.82 | 1.03 (0.84-1.26) | 1.14 (0.76-1.70) | 0.57 |
|  | rs2285332 | 7 | 92,079,569 | 3' UTR | G>C | 1.00 | 0.24 | 0.27 | 1.02 (0.83-1.25) | 1.23 (0.80-1.89) | 0.48 |
|  | rs42038 | 7 | 92,081,655 | 3' UTR | G>A | 1.00 | 0.30 | 0.26 | 1.13 (0.92-1.39) | 1.21 (0.87-1.69) | 0.15 |
|  | rs42039 | 7 | 92,082,358 | 3' UTR | G>A | 1.00 | 0.24 | 0.51 | 1.12 (0.91-1.38) | 1.18 (0.79-1.76) | 0.22 |
|  | rs42041 | 7 | 92,084,680 | intron | G>C | 0.98 | 0.25 | 0.44 | 1.09 (0.88-1.33) | 1.14 (0.77-1.70) | 0.36 |
|  | rs42042 | 7 | 92,085,048 | intron | A>G | 0.98 | 0.51 | 0.77 | 0.91 (0.71-1.15) | 0.82 (0.62-1.08) | 0.17 |
|  | rs42235 | 7 | 92,086,012 | intron | G>A | 0.98 | 0.29 | 0.15 | 1.14 (0.92-1.40) | 1.19 (0.85-1.67) | 0.17 |
|  | rs191777 | 7 | 92,088,310 | intron | A>T | 0.98 | 0.48 | 0.57 | 0.86 (0.68-1.08) | 0.89 (0.67-1.17) | 0.36 |
|  | rs3731348 | 7 | 92,091,169 | intron | C>A | 0.98 | 0.05 | 0.69 | 1.05 (0.76-1.45) | 1.99 (0.35-11.4) | 0.57 |
|  | rs2237570 | 7 | 92,096,190 | intron | A>T | 0.98 | 0.10 | 0.09 | 1.08 (0.85-1.39) | 1.41 (0.41-4.79) | 0.43 |
|  | rs17688839 | 7 | 92,101,286 | intron | A>T | 0.98 | 0.19 | 0.04 | 1.21 (0.97-1.50) | 0.87 (0.53-1.44) | 0.36 |
|  | rs2282978 | 7 | 92,102,346 | intron | A>G | 0.98 | 0.33 | 0.15 | 1.12 (0.91-1.38) | 1.20 (0.88-1.64) | 0.18 |
|  | rs2282979 | 7 | 92,102,929 | intron | A>G | 0.98 | 0.28 | 0.11 | 1.16 (0.95-1.43) | 1.07 (0.74-1.53) | 0.29 |
|  | rs10225965 | 7 | 92,111,514 | intron | G>A | 0.98 | 0.19 | 0.43 | 0.96 (0.78-1.19) | 1.25 (0.74-2.12) | 0.84 |
|  | rs2282983 | 7 | 92,117,299 | intron | A>G | 0.98 | 0.35 | 0.05 | 1.19 (0.96-1.47) | 1.27 (0.94-1.71) | 0.07 |
|  | rs11773884 | 7 | 92,123,059 | intron | A>G | 0.98 | 0.30 | 0.05 | 1.18 (0.96-1.45) | 1.15 (0.83-1.61) | 0.16 |
|  | rs9640606 | 7 | 92,138,976 | intron | G>A | 0.98 | 0.09 | 0.97 | 1.16 (0.90-1.50) | 0.83 (0.26-2.65) | 0.36 |
|  | rs12670783 | 7 | 92,146,734 | intron | A>G | 0.98 | 0.10 | 0.64 | 1.13 (0.88-1.45) | 0.84 (0.30-2.31) | 0.49 |
|  | rs7781436 | 7 | 92,155,688 | intron | A>G | 0.98 | 0.22 | 0.52 | 0.87 (0.71-1.08) | 0.57 (0.34-0.95) | 0.03 |
|  | rs2282990 | 7 | 92,164,404 | intron | G>A | 0.91 | 0.07 | 0.81 | 1.48 (1.11-1.98) | 1.03 (0.26-4.01) | 0.01 |
|  | rs3731321 | 7 | 92,165,701 | intron | A>G | 0.98 | 0.19 | 0.51 | 0.82 (0.66-1.01) | 1.21 (0.70-2.07) | 0.32 |
|  | rs2079147 | 7 | 92,170,311 | intron | A>G | 0.98 | 0.54 | 0.95 | 0.88 (0.69-1.13) | 0.74 (0.56-0.98) | 0.03 |
|  | rs2282991 | 7 | 92,183,017 | intron | A>T | 0.98 | 0.08 | 0.91 | 1.26 (0.97-1.65) | 0.73 (0.20-2.68) | 0.15 |
|  | rs17690388 | 7 | 92,183,293 | intron | G>A | 0.98 | 0.07 | 0.85 | 0.75 (0.55-1.01) | 1.71 (0.42-6.86) | 0.15 |
|  | rs2374594 | 7 | 92,235,227 | intron | G>A | 0.98 | 0.08 | 0.01 | 1.29 (0.97-1.71) | 0.61 (0.20-1.82) | 0.25 |
|  | rs445 | 7 | 92,246,306 | intron | G>A | 0.98 | 0.11 | 0.16 | 0.97 (0.76-1.23) | 0.89 (0.28-2.77) | 0.74 |
|  | rs1005346 | 7 | 92,261,441 | intron | G>A | 0.98 | 0.30 | 0.71 | 1.30 (1.06-1.60) | 1.25 (0.88-1.78) | 0.03 |
|  | rs6975474 | 7 | 92,273,122 | intron | G>A | 0.98 | 0.06 | 0.90 | 1.19 (0.88-1.60) | 3.14 (0.90-10.9) | 0.07 |
|  | rs3731267 | 7 | 92,298,611 | intron | G>A | 0.46 | 0.14 | 0.60 | 1.22 (0.87-1.70) | 0.80 (0.29-2.22) | 0.48 |
| *CUL1* | rs243552 | 7 | 148,024,030 | 5' UTR | G>C | 0.98 | 0.43 | 0.04 | 1.07 (0.86-1.33) | 0.82 (0.61-1.08) | 0.28 |
|  | rs122571 | 7 | 148,025,528 | 5' UTR | A>G | 0.98 | 0.41 | 0.01 | 1.22 (0.98-1.52) | 0.96 (0.73-1.27) | 0.84 |
|  | rs243551 | 7 | 148,026,057 | 5' UTR | C>G | 0.98 | 0.40 | 0.06 | 1.23 (0.99-1.53) | 0.91 (0.68-1.22) | 1.00 |
|  | rs10235843 | 7 | 148,031,399 | intron | A>C | 0.98 | 0.03 | 0.47 | 0.97 (0.62-1.52) | - | 0.77 |
|  | rs243538 | 7 | 148,035,312 | intron | G>A | 0.98 | 0.27 | 0.08 | 1.12 (0.91-1.38) | 0.78 (0.53-1.13) | 0.80 |
|  | rs243529 | 7 | 148,044,734 | intron | G>A | 0.98 | 0.35 | 0.14 | 1.14 (0.92-1.40) | 0.98 (0.72-1.33) | 0.70 |
|  | rs10488072 | 7 | 148,049,999 | intron | C>T | 1.00 | 0.06 | 0.38 | 1.31 (0.98-1.76) | 0.46 (0.09-2.42) | 0.17 |
|  | rs243523 | 7 | 148,050,417 | intron | T>G | 1.00 | 0.30 | 1.00 | 0.90 (0.73-1.10) | 0.95 (0.67-1.34) | 0.44 |
|  | rs11760399 | 7 | 148,053,514 | intron | A>G | 0.98 | 0.08 | 0.21 | 1.03 (0.79-1.36) | 2.03 (0.47-8.88) | 0.58 |
|  | rs243510 | 7 | 148,063,467 | intron | A>G | 0.98 | 0.35 | 0.13 | 1.08 (0.88-1.33) | 0.96 (0.70-1.30) | 0.93 |
|  | rs243503 | 7 | 148,067,039 | intron | C>G | 1.00 | 0.35 | 0.15 | 1.08 (0.88-1.33) | 0.97 (0.71-1.32) | 0.88 |
|  | rs243492 | 7 | 148,076,741 | intron | G>A | 1.00 | 0.47 | 0.18 | 1.16 (0.92-1.47) | 1.12 (0.84-1.48) | 0.41 |
|  | rs243491 | 7 | 148,077,106 | intron | A>G | 0.98 | 0.49 | 0.23 | 1.08 (0.85-1.37) | 1.06 (0.80-1.40) | 0.70 |
|  | rs243487 | 7 | 148,079,144 | intron | G>A | 1.00 | 0.28 | 0.14 | 1.07 (0.87-1.31) | 0.80 (0.55-1.15) | 0.65 |
|  | rs243486 | 7 | 148,079,154 | intron | A>C | 0.98 | 0.28 | 0.10 | 1.10 (0.89-1.35) | 0.81 (0.56-1.18) | 0.80 |
|  | rs243482 | 7 | 148,081,454 | intron | T>G | 1.00 | 0.50 | 0.20 | 1.03 (0.82-1.31) | 1.06 (0.80-1.40) | 0.67 |
|  | rs243480 | 7 | 148,083,838 | intron | C>A | 0.98 | 0.28 | 0.10 | 1.08 (0.87-1.32) | 0.79 (0.54-1.14) | 0.64 |
|  | rs10246773 | 7 | 148,091,469 | intron | A>G | 0.98 | 0.04 | 0.33 | 1.00 (0.68-1.47) | 2.70 (0.46-15.9) | 0.64 |
|  | rs17537406 | 7 | 148,093,110 | intron | A>G | 0.98 | 0.04 | 0.42 | 1.34 (0.96-1.88) | 0.77 (0.13-4.75) | 0.13 |
|  | rs1014095 | 7 | 148,094,793 | intron | G>A | 1.00 | 0.24 | 0.10 | 1.12 (0.91-1.38) | 0.87 (0.58-1.31) | 0.78 |
|  | rs3823635 | 7 | 148,096,921 | intron | C>T | 1.00 | 0.46 | 0.38 | 1.00 (0.80-1.26) | 1.03 (0.78-1.36) | 0.83 |
|  | rs3779033 | 7 | 148,097,051 | intron | G>C | 0.98 | 0.05 | 0.38 | 1.38 (1.01-1.87) | 0.56 (0.10-3.12) | 0.10 |
|  | rs3807446 | 7 | 148,098,380 | intron | A>G | 0.98 | 0.05 | 0.77 | 0.93 (0.66-1.29) | 3.25 (0.59-17.8) | 0.90 |
|  | rs1029888 | 7 | 148,100,649 | intron | A>G | 0.98 | 0.24 | 0.25 | 1.12 (0.91-1.38) | 0.91 (0.60-1.38) | 0.68 |
|  | rs3779035 | 7 | 148,101,431 | intron | A>G | 1.00 | 0.48 | 0.45 | 0.94 (0.75-1.19) | 1.00 (0.76-1.31) | 0.97 |
|  | rs6958001 | 7 | 148,105,685 | intron | G>A | Monomorphic | - | - | - | - | - |
|  | rs17171109 | 7 | 148,106,297 | intron | T>C | 1.00 | 0.07 | 0.21 | 1.13 (0.86-1.50) | 3.61 (0.71-18.4) | 0.17 |
|  | rs1558420 | 7 | 148,111,047 | intron | A>C | 1.00 | 0.3 | 0.63 | 1.07 (0.88-1.32) | 1.24 (0.88-1.75) | 0.21 |
|  | rs17853287 | 7 | 148,111,848 | ns K375R | T>C | Failed | - | - | - | - | - |
|  | rs10271133 | 7 | 148,111,923 | intron | G>A | 1.00 | 0.24 | 0.08 | 0.92 (0.75-1.13) | 0.92 (0.62-1.35) | 0.44 |
|  | rs7778273 | 7 | 148,115,911 | intron | G>A | 0.98 | 0.07 | 0.22 | 1.14 (0.86-1.51) | 3.57 (0.70-18.2) | 0.17 |
|  | rs2058388 | 7 | 148,117,355 | intron | G>C | 1.00 | 0.28 | 0.23 | 1.00 (0.82-1.22) | 1.24 (0.85-1.80) | 0.45 |
|  | rs17855577 | 7 | 148,117,826 | ns S550F | C>T | Failed | - | - | - | - | - |
|  | rs1019218 | 7 | 148,119,897 | intron | A>G | 0.98 | 0.36 | 0.71 | 1.07 (0.87-1.32) | 1.08 (0.80-1.47) | 0.50 |
|  | rs17171113 | 7 | 148,120,753 | synon | G>A | Failed | - | - | - | - | - |
|  | rs3807449 | 7 | 148,124,977 | intron | A>G | 1.00 | 0.16 | 0.40 | 0.86 (0.69-1.08) | 0.95 (0.53-1.70) | 0.28 |
|  | rs12154650 | 7 | 148,130,274 | 3' UTR | C>G | 0.97 | 0.04 | 1.00 | 1.04 (0.73-1.48) | 4.55 (0.40-51.6) | 0.56 |
| *E2F5* | rs2896452 | 8 | 86,289,475 | intron | T>C | 1.00 | 0.23 | 0.88 | 1.15 (0.94-1.41) | 0.82 (0.52-1.29) | 0.65 |
|  | rs4150893 | 8 | 86,292,006 | intron | G>A | 1.00 | 0.29 | 0.65 | 1.21 (0.99-1.48) | 0.90 (0.63-1.29) | 0.54 |
|  | rs4150895 | 8 | 86,292,487 | intron | A>C | Failed | - | - | - | - | - |
|  | rs4150909 | 8 | 86,295,088 | intron | T>C | 1.00 | 0.41 | 0.96 | 1.02 (0.83-1.27) | 0.93 (0.69-1.24) | 0.70 |
|  | rs2403083 | 8 | 86,295,401 | intron | T>G | 1.00 | 0.24 | 0.94 | 1.12 (0.92-1.37) | 0.83 (0.54-1.29) | 0.81 |
|  | rs3808538 | 8 | 86,308,563 | intron | T>G | 1.00 | 0.24 | 0.94 | 1.16 (0.95-1.42) | 0.91 (0.59-1.39) | 0.49 |
| *CCNE2* | rs1129152 | 8 | 95,957,483 | 3' (4,145) | C>T | 1.00 | 0.40 | 0.83 | 1.05 (0.85-1.30) | 1.10 (0.82-1.47) | 0.50 |
|  | rs716733 | 8 | 95,958,157 | 3' (3,471) | C>T | 1.00 | 0.23 | 0.18 | 0.89 (0.72-1.09) | 0.84 (0.52-1.35) | 0.21 |
|  | rs6983000 | 8 | 95,960,159 | 3' (1,469) | A>T | 1.00 | 0.30 | 0.45 | 1.13 (0.92-1.38) | 0.94 (0.66-1.34) | 0.69 |
|  | rs12679044 | 8 | 95,960,532 | 3' (1,096) | C>A | 1.00 | 0.51 | 0.21 | 0.97 (0.76-1.23) | 1.07 (0.81-1.42) | 0.62 |
|  | rs11775475 | 8 | 95,974,665 | intron | A>C | 1.00 | 0.19 | 0.14 | 0.91 (0.74-1.13) | 0.96 (0.53-1.72) | 0.45 |
|  | rs2466431 | 8 | 95,975,730 | intron | C>G | 1.00 | 0.05 | 0.69 | 1.00 (0.72-1.37) | 0.55 (0.05-6.23) | 0.88 |
|  | rs550564 | 8 | 95,980,925 | 5' (4,265) | C>T | 1.00 | 0.27 | 0.51 | 1.11 (0.90-1.36) | 1.03 (0.71-1.50) | 0.49 |
| *CDKN2A* | rs3731249 | 9 | 21,960,916 | 3' UTR | G>A | 1.00 | 0.04 | 0.11 | 1.05 (0.70-1.55) | 0.31 (0.03-3.31) | 0.88 |
|  | rs2811709 | 9 | 21,970,151 | intron | G>A | 1.00 | 0.14 | 0.29 | 0.88 (0.70-1.11) | 0.49 (0.22-1.09) | 0.07 |
|  | rs7036656 | 9 | 21,980,457 | intron | A>G | 1.00 | 0.29 | 0.33 | 0.88 (0.72-1.08) | 0.72 (0.50-1.04) | 0.06 |
| *CDKN2B* | rs3218009 | 9 | 21,988,757 | 3' (4,145) | C>G | 1.00 | 0.12 | 0.28 | 1.08 (0.86-1.36) | 1.88 (0.86-4.09) | 0.19 |
|  | rs1063192 | 9 | 21,993,367 | 3' UTR | T>C | 1.00 | 0.43 | 0.70 | 1.06 (0.85-1.32) | 1.18 (0.89-1.55) | 0.26 |
|  | rs2069418 | 9 | 21,999,698 | 5' (386) | G>C | 1.00 | 0.45 | 1.00 | 1.03 (0.82-1.29) | 1.25 (0.95-1.64) | 0.13 |
|  | rs575427 | 9 | 22,001,477 | 5' (2,165) | A>G | 1.00 | 0.10 | 0.86 | 0.89 (0.69-1.15) | 1.02 (0.38-2.73) | 0.44 |
|  | rs13298881 | 9 | 22,002,051 | 5' (2,739) | A>G | 1.00 | 0.10 | 0.94 | 0.92 (0.72-1.19) | 1.47 (0.61-3.51) | 0.90 |
|  | rs10811640 | 9 | 22,003,411 | 5' (4,099) | C>A | 1.00 | 0.44 | 0.49 | 0.91 (0.73-1.14) | 0.93 (0.70-1.22) | 0.52 |
| *ABL1* | rs7020059 | 9 | 132,588,405 | intron | C>T | 1.00 | 0.11 | 0.84 | 1.09 (0.86-1.38) | 0.89 (0.36-2.21) | 0.61 |
|  | rs7024632 | 9 | 132,592,262 | intron | T>C | 1.00 | 0.39 | 0.09 | 1.01 (0.81-1.25) | 1.25 (0.93-1.68) | 0.22 |
|  | rs10751508 | 9 | 132,597,937 | intron | C>T | Failed | - | - | - | - | - |
|  | rs2789769 | 9 | 132,604,796 | intron | G>A | 1.00 | 0.26 | 0.29 | 0.98 (0.80-1.21) | 1.37 (0.93-2.02) | 0.34 |
|  | rs11244120 | 9 | 132,617,316 | intron | C>A | 0.99 | 0.13 | 0.64 | 1.12 (0.89-1.40) | 1.06 (0.51-2.21) | 0.38 |
|  | rs4740363 | 9 | 132,619,005 | intron | A>G | 1.00 | 0.06 | 0.38 | 0.97 (0.71-1.31) | 0.89 (0.20-3.90) | 0.80 |
|  | rs2855171 | 9 | 132,628,764 | intron | G>A | 1.00 | 0.30 | 0.46 | 0.92 (0.75-1.12) | 1.01 (0.71-1.43) | 0.66 |
|  | rs2791743 | 9 | 132,630,598 | intron | A>T | 1.00 | 0.23 | 0.75 | 0.99 (0.81-1.21) | 0.86 (0.54-1.35) | 0.63 |
|  | rs2855172 | 9 | 132,641,469 | intron | G>T | 1.00 | 0.44 | 0.50 | 0.94 (0.76-1.18) | 0.91 (0.69-1.19) | 0.47 |
|  | rs10901285 | 9 | 132,660,695 | intron | G>A | 1.00 | 0.29 | 0.05 | 1.08 (0.88-1.32) | 1.08 (0.77-1.50) | 0.50 |
|  | rs10901291 | 9 | 132,707,702 | intron | G>A | 1.00 | 0.16 | 0.39 | 1.05 (0.85-1.31) | 0.81 (0.45-1.47) | 0.99 |
|  | rs2855192 | 9 | 132,717,872 | intron | C>T | 1.00 | 0.12 | 0.40 | 1.14 (0.90-1.44) | 2.81 (1.29-6.09) | 0.02 |
|  | rs11788639 | 9 | 132,719,994 | intron | A>C | Failed | - | - | - | - | - |
|  | rs3824400 | 9 | 132,723,896 | intron | C>T | 1.00 | 0.13 | 0.32 | 1.08 (0.86-1.36) | 0.94 (0.47-1.85) | 0.68 |
|  | rs3780282 | 9 | 132,733,475 | intron | G>A | 1.00 | 0.15 | 0.68 | 1.08 (0.87-1.34) | 1.03 (0.55-1.91) | 0.56 |
|  | rs2696141 | 9 | 132,743,920 | intron | C>A | 1.00 | 0.10 | 0.62 | 1.04 (0.81-1.33) | 0.54 (0.18-1.61) | 0.83 |
|  | rs4740377 | 9 | 132,744,041 | intron | T>C | 0.99 | 0.20 | 0.95 | 0.95 (0.77-1.17) | 0.73 (0.44-1.23) | 0.30 |
|  | rs3808815 | 9 | 132,747,516 | intron | G>A | 1.00 | 0.10 | 0.98 | 0.87 (0.67-1.12) | 0.54 (0.19-1.55) | 0.15 |
|  | rs1056209 | 9 | 132,752,411 | 3' UTR | G>A | 0.99 | 0.08 | 0.57 | 1.03 (0.78-1.36) | 0.37 (0.07-1.80) | 0.80 |
| *CDC2* | rs2127355 | 10 | 62,204,222 | 5' (1,468) | A>G | 1.00 | 0.47 | 0.23 | 1.08 (0.86-1.35) | 1.00 (0.76-1.32) | 0.93 |
|  | rs3213025 | 10 | 62,209,197 | intron | A>G | 1.00 | 0.16 | 0.88 | 1.01 (0.81-1.26) | 1.05 (0.58-1.92) | 0.88 |
|  | rs2448343 | 10 | 62,209,650 | intron | G>A | 1.00 | 0.38 | 0.02 | 1.13 (0.91-1.39) | 0.71 (0.52-0.97) | 0.20 |
|  | rs2260179 | 10 | 62,209,794 | intron | G>C | 1.00 | 0.32 | 0.01 | 1.26 (1.02-1.54) | 0.85 (0.60-1.18) | 0.71 |
|  | rs3213046 | 10 | 62,214,864 | intron | A>G | 1.00 | 0.32 | 0.79 | 1.11 (0.90-1.36) | 1.10 (0.79-1.53) | 0.38 |
|  | rs2448347 | 10 | 62,215,148 | intron | C>T | 1.00 | 0.43 | 0.74 | 1.02 (0.82-1.27) | 1.06 (0.80-1.40) | 0.69 |
|  | rs2170007 | 10 | 62,217,575 | intron | T>G | 1.00 | 0.27 | 0.63 | 0.98 (0.80-1.20) | 0.69 (0.46-1.04) | 0.21 |
|  | rs3213077 | 10 | 62,222,738 | intron | T>C | 1.00 | 0.19 | 0.59 | 0.96 (0.78-1.19) | 0.95 (0.58-1.58) | 0.70 |
|  | rs1871446 | 10 | 62,223,769 | 3' UTR | G>A | 1.00 | 0.25 | 0.41 | 0.96 (0.79-1.18) | 0.65 (0.42-1.01) | 0.15 |
|  | rs10711 | 10 | 62,224,480 | 3' (550) | A>C | Failed | - | - | - | - | - |
|  | rs16915552 | 10 | 62,227,143 | 3' (3,213) | G>A | 1.00 | 0.12 | 0.17 | 0.94 (0.74-1.20) | 0.68 (0.31-1.47) | 0.35 |
| *CCND1* | rs9344 | 11 | 69,172,091 | synon | C>T | 1.00 | 0.46 | 0.03 | 1.23 (0.98-1.53) | 1.04 (0.79-1.37) | 0.62 |
|  | rs649392 | 11 | 69,173,974 | intron | T>C | 1.00 | 0.46 | 0.06 | 1.07 (0.86-1.34) | 0.92 (0.70-1.21) | 0.66 |
|  | rs678653 | 11 | 69,175,918 | 3' UTR | C>G | 1.00 | 0.35 | 0.26 | 1.08 (0.88-1.33) | 0.93 (0.69-1.27) | 0.97 |
|  | rs7178 | 11 | 69,178,211 | 3' UTR | T>C | 1.00 | 0.09 | 0.29 | 0.82 (0.63-1.07) | 0.72 (0.17-3.12) | 0.14 |
|  | rs11603541 | 11 | 69,181,554 | 3' (3,131) | G>C | 1.00 | 0.12 | 0.31 | 0.90 (0.71-1.15) | 0.63 (0.22-1.80) | 0.27 |
| *CCND2* | rs1049606 | 12 | 4,253,297 | 5' UTR | T>C | 1.00 | 0.42 | 0.80 | 1.13 (0.91-1.40) | 1.16 (0.87-1.55) | 0.25 |
|  | rs3217926 | 12 | 4,281,944 | 3' UTR | A>G | 1.00 | 0.38 | 0.29 | 0.95 (0.77-1.17) | 1.17 (0.86-1.58) | 0.53 |
|  | rs1049612 | 12 | 4,283,023 | 3' UTR | A>G | 1.00 | 0.38 | 0.35 | 0.96 (0.78-1.18) | 1.16 (0.86-1.57) | 0.52 |
|  | rs3217933 | 12 | 4,283,261 | 3' UTR | T>C | 1.00 | 0.26 | 0.42 | 0.98 (0.80-1.20) | 0.89 (0.59-1.34) | 0.62 |
| *CDKN1B* | rs3759216 | 12 | 12,759,353 | 5' (2,223) | C>T | 1.00 | 0.44 | 0.19 | 0.98 (0.79-1.23) | 1.16 (0.87-1.53) | 0.38 |
|  | rs3759217 | 12 | 12,759,719 | 5' (1,857) | C>T | 1.00 | 0.12 | 0.53 | 0.97 (0.76-1.24) | 1.32 (0.65-2.68) | 0.83 |
|  | rs34330 | 12 | 12,761,962 | 5' UTR | C>T | 1.00 | 0.25 | 0.28 | 1.01 (0.82-1.24) | 0.78 (0.52-1.17) | 0.47 |
|  | rs34329 | 12 | 12,764,500 | intron | C>G | 1.00 | 0.30 | 0.43 | 0.92 (0.75-1.13) | 1.31 (0.93-1.84) | 0.47 |
|  | rs3093736 | 12 | 12,764,568 | intron | C>T | 1.00 | 0.03 | 0.61 | 1.00 (0.67-1.49) | 1.04 (0.05-20.2) | 0.99 |
|  | rs7330 | 12 | 12,766,184 | 3' UTR | T>G | 1.00 | 0.40 | 0.64 | 0.98 (0.79-1.21) | 1.06 (0.80-1.42) | 0.76 |
|  | rs1420023 | 12 | 12,767,378 | 3' (808) | G>C | 1.00 | 0.13 | 0.74 | 0.89 (0.70-1.13) | 0.79 (0.36-1.73) | 0.27 |
|  | rs34322 | 12 | 12,770,837 | 3' (4,267) | C>T | 1.00 | 0.48 | 0.41 | 0.91 (0.72-1.14) | 0.98 (0.74-1.29) | 0.82 |
| *CDK2* | rs2069391 | 12 | 54,646,145 | 5' (681) | C>T | 1.00 | 0.07 | 0.56 | 1.15 (0.88-1.51) | 1.38 (0.35-5.50) | 0.27 |
|  | rs2069408 | 12 | 54,650,588 | intron | A>G | 0.99 | 0.33 | 0.54 | 0.98 (0.80-1.21) | 0.90 (0.65-1.24) | 0.57 |
|  | rs2069414 | 12 | 54,651,966 | 3' UTR | G>T | 0.99 | 0.06 | 0.44 | 1.36 (1.02-1.81) | 1.70 (0.27-10.83) | 0.03 |
|  | rs1045435 | 12 | 54,652,427 | 3' UTR | C>G | 1.00 | 0.08 | 0.48 | 1.31 (1.01-1.71) | 0.39 (0.07-2.14) | 0.11 |
|  | rs11171710 | 12 | 54,654,345 | 3' (1,510) | G>A | 1.00 | 0.45 | 0.15 | 1.04 (0.83-1.30) | 1.01 (0.77-1.33) | 0.89 |
|  | rs17528736 | 12 | 54,654,785 | 3' (1,950) | C>T | 1.00 | 0.03 | 1.00 | 1.23 (0.84-1.80) | 0.73 (0.04-12.2) | 0.33 |
|  | rs773108 | 12 | 54,656,178 | 3' (3,343) | T>C | 1.00 | 0.33 | 0.53 | 1.03 (0.84-1.26) | 0.85 (0.61-1.18) | 0.54 |
| *CDK4* | rs2069502 | 12 | 56,430,932 | intron | G>A | 1.00 | 0.33 | 0.12 | 1.02 (0.83-1.25) | 0.96 (0.70-1.32) | 0.93 |
|  | rs2270777 | 12 | 56,431,423 | intron | G>A | 1.00 | 0.43 | 0.13 | 0.99 (0.80-1.24) | 0.78 (0.59-1.03) | 0.13 |
|  | rs2072052 | 12 | 56,432,986 | 5' (555) | T>G | 1.00 | 0.33 | 0.15 | 1.01 (0.82-1.25) | 1.00 (0.73-1.37) | 0.97 |
| *CCNA1* | rs9576046 | 13 | 35,900,787 | 5' (3,846) | A>T | 1.00 | 0.18 | 0.02 | 0.92 (0.73-1.14) | 0.68 (0.39-1.17) | 0.16 |
|  | rs7985423 | 13 | 35,904,463 | 5' (170) | G>C | 1.00 | 0.26 | 0.94 | 1.13 (0.92-1.38) | 1.29 (0.88-1.90) | 0.11 |
|  | rs2282411 | 13 | 35,912,366 | intron | G>A | 1.00 | 0.40 | 0.75 | 1.06 (0.85-1.31) | 1.13 (0.85-1.50) | 0.41 |
|  | rs17188012 | 13 | 35,913,242 | intron | G>C | 1.00 | 0.11 | 0.40 | 0.92 (0.72-1.18) | 1.66 (0.66-4.15) | 0.92 |
|  | rs11147610 | 13 | 35,917,186 | 3' (2,178) | A>G | 1.00 | 0.43 | 0.54 | 1.09 (0.88-1.36) | 1.06 (0.80-1.40) | 0.59 |
| *RB1* | rs4151467 | 13 | 47,817,924 | intron | T>C | 1.00 | 0.05 | 0.80 | 1.08 (0.78-1.48) | 2.21 (0.50-9.77) | 0.41 |
|  | rs7329938 | 13 | 47,829,980 | intron | A>G | 1.00 | 0.12 | 0.49 | 1.12 (0.88-1.42) | 1.16 (0.55-2.46) | 0.33 |
|  | rs4151510 | 13 | 47,843,176 | intron | C>T | 1.00 | 0.13 | 0.50 | 0.99 (0.79-1.25) | 0.99 (0.44-2.26) | 0.93 |
|  | rs9568036 | 13 | 47,869,937 | intron | T>C | 1.00 | 0.40 | 0.96 | 1.05 (0.85-1.31) | 1.02 (0.76-1.37) | 0.79 |
|  | rs198604 | 13 | 47,882,064 | intron | C>T | 1.00 | 0.24 | 0.71 | 1.02 (0.83-1.25) | 1.10 (0.72-1.66) | 0.68 |
|  | rs4151551 | 13 | 47,883,440 | intron | G>T | 1.00 | 0.09 | 0.24 | 1.13 (0.87-1.46) | 2.36 (0.66-8.50) | 0.19 |
|  | rs2854344 | 13 | 47,895,694 | intron | G>A | 1.00 | 0.07 | 0.15 | 1.05 (0.78-1.41) | 0.76 (0.23-2.49) | 0.95 |
|  | rs990814 | 13 | 47,957,578 | 3' (3,551) | T>C | 1.00 | 0.28 | 0.79 | 1.08 (0.88-1.32) | 1.05 (0.72-1.53) | 0.55 |
| *TFDP1* | rs11617870 | 13 | 113,289,228 | intron | T>C | Failed | - | - | - | - | - |
|  | rs9577581 | 13 | 113,300,003 | intron | C>T | 1.00 | 0.23 | 0.65 | 0.92 (0.75-1.13) | 0.85 (0.54-1.35) | 0.33 |
|  | rs4415916 | 13 | 113,309,352 | intron | T>G | Failed | - | - | - | - | - |
|  | rs4150724 | 13 | 113,312,518 | intron | C>T | 1.00 | 0.06 | 0.42 | 1.21 (0.90-1.62) | 2.51 (0.47-13.3) | 0.12 |
|  | rs4150729 | 13 | 113,313,306 | intron | G>A | 1.00 | 0.05 | 0.63 | 0.92 (0.67-1.28) | - | 0.50 |
|  | rs4150734 | 13 | 113,313,951 | intron | T>C | 0.99 | 0.37 | 0.09 | 0.97 (0.79-1.20) | 1.08 (0.79-1.48) | 0.79 |
|  | rs4494444 | 13 | 113,326,560 | intron | A>C | 1.00 | 0.38 | 0.76 | 1.00 (0.81-1.24) | 1.06 (0.79-1.42) | 0.76 |
|  | rs12867405 | 13 | 113,331,064 | intron | C>T | 1.00 | 0.23 | 0.27 | 0.97 (0.79-1.19) | 1.00 (0.66-1.52) | 0.84 |
|  | rs4150828 | 13 | 113,342,809 | 3' UTR | C>G | 1.00 | 0.06 | 0.45 | 0.99 (0.72-1.34) | 0.64 (0.06-7.23) | 0.86 |
| *CCNB2* | rs11631644 | 15 | 57,180,303 | 5' (4,309) | C>A | 1.00 | 0.11 | 0.90 | 0.93 (0.72-1.19) | 1.00 (0.41-2.44) | 0.60 |
|  | rs17302191 | 15 | 57,195,920 | intron | A>G | 1.00 | 0.31 | 0.91 | 1.06 (0.87-1.31) | 1.22 (0.88-1.69) | 0.25 |
|  | rs1486878 | 15 | 57,202,759 | intron | C>G | 1.00 | 0.27 | 0.20 | 1.04 (0.85-1.27) | 1.53 (1.06-2.21) | 0.08 |
| *PLK1* | rs27770 | 16 | 23,609,039 | 3' UTR | A>G | 0.48 | 0.20 | 0.62 | 1.21 (0.89-1.64) | 0.91 (0.43-1.89) | 0.45 |
| *RBL2* | rs8056349 | 16 | 52,025,527 | 5' (374) | G>C | 1.00 | 0.44 | 0.79 | 0.99 (0.79-1.24) | 1.25 (0.95-1.65) | 0.14 |
|  | rs16952242 | 16 | 52,031,751 | intron | G>A | 0.99 | 0.29 | 0.54 | 1.08 (0.89-1.33) | 1.21 (0.85-1.73) | 0.24 |
|  | rs17800727 | 16 | 52,038,511 | ns Y210C | A>G | 1.00 | 0.29 | 0.68 | 1.12 (0.92-1.37) | 1.19 (0.83-1.70) | 0.20 |
|  | rs13329946 | 16 | 52,045,418 | intron | G>A | 1.00 | 0.07 | 0.28 | 0.98 (0.73-1.31) | 3.22 (0.56-18.5) | 0.77 |
|  | rs17801093 | 16 | 52,053,682 | intron | C>A | 1.00 | 0.14 | 0.49 | 0.96 (0.76-1.20) | 1.44 (0.72-2.88) | 0.85 |
|  | rs3929 | 16 | 52,081,809 | 3' UTR | G>C | 1.00 | 0.44 | 0.98 | 1.02 (0.81-1.27) | 1.26 (0.96-1.66) | 0.13 |
| *E2F4* | rs11700 | 16 | 65,790,185 | 3' UTR | T>C | 0.99 | 0.07 | 0.87 | 1.04 (0.78-1.39) | 0.64 (0.15-2.74) | 0.98 |
| *CDKN2D* | rs1465701 | 19 | 10,539,842 | intron | G>A | 1.00 | 0.25 | 0.81 | 1.05 (0.86-1.29) | 1.28 (0.86-1.91) | 0.27 |
|  | rs17677316 | 19 | 10,541,241 | 5' (610) | G>A | 1.00 | 0.25 | 0.29 | 0.85 (0.70-1.05) | 0.95 (0.62-1.46) | 0.25 |
| *CCNE1* | rs997669 | 19 | 34,996,323 | intron | T>C | 1.00 | 0.39 | 0.54 | 0.88 (0.71-1.09) | 0.91 (0.68-1.23) | 0.36 |
|  | rs1406 | 19 | 35,006,952 | 3' UTR | C>A | 0.99 | 0.23 | 0.34 | 0.98 (0.80-1.20) | 1.11 (0.69-1.77) | 0.91 |
| *CDC25B* | rs1010608 | 20 | 3,732,593 | intron | C>T | 1.00 | 0.24 | 0.98 | 0.96 (0.78-1.18) | 1.04 (0.68-1.57) | 0.88 |
| *E2F1* | rs3213183 | 20 | 31,726,623 | 3' (527) | G>A | 1.00 | 0.29 | 0.01 | 1.09 (0.89-1.33) | 0.88 (0.62-1.24) | 0.90 |
|  | rs3213182 | 20 | 31,726,894 | 3' (256) | A>C | Failed | - | - | - | - | - |
|  | rs2071056 | 20 | 31,729,174 | intron | T>C | 1.00 | 0.29 | 0.01 | 1.10 (0.89-1.35) | 0.93 (0.66-1.30) | 0.89 |
| *RBL1* | rs6030712 | 20 | 35,070,812 | intron | G>A | 1.00 | 0.14 | 0.12 | 0.98 (0.78-1.23) | 0.68 (0.35-1.32) | 0.44 |
|  | rs3897903 | 20 | 35,106,470 | intron | C>T | 1.00 | 0.23 | 0.49 | 1.05 (0.85-1.29) | 0.83 (0.54-1.28) | 0.83 |
|  | rs6093874 | 20 | 35,125,339 | intron | A>G | 1.00 | 0.50 | 0.95 | 0.99 (0.78-1.25) | 1.11 (0.85-1.46) | 0.42 |

Multivariate logistic regression analyses were adjusted for age (<40, 40-49, 50-59, 60-69, 70+ years), site (Minnesota, Wisconsin, Illinois, North Dakota, South Dakota, North Carolina), oral contraceptive use, hormone therapy, parity and body mass index.
